# Mechanisms of ventricular arrhythmias elicited by coexistence of multiple electrophysiological remodeling in ischemia: A simulation study

**DOI:** 10.1371/journal.pcbi.1009388

**Published:** 2022-04-27

**Authors:** Cuiping Liang, Qince Li, Kuanquan Wang, Yimei Du, Wei Wang, Henggui Zhang

**Affiliations:** 1 School of Computer Science and Technology, Harbin Institute of Technology (HIT), Harbin, China; 2 Peng Cheng Laboratory, Shenzhen, China; 3 Wuhan Union Hospital, Tongji Medical College of Huazhong University of Science and Technology, Wuhan, China; 4 School of Physics and Astronomy, The University of Manchester, Manchester, United Kingdom; 5 Key Laboratory of Medical Electrophysiology of Ministry of Education and Medical Electrophysiological Key Laboratory of Sichuan Province, Institute of Cardiovascular Research, Southwest Medical University, Luzhou, China; University of California San Diego, UNITED STATES

## Abstract

Myocardial ischemia, injury and infarction (MI) are the three stages of acute coronary syndrome (ACS). In the past two decades, a great number of studies focused on myocardial ischemia and MI individually, and showed that the occurrence of reentrant arrhythmias is often associated with myocardial ischemia or MI. However, arrhythmogenic mechanisms in the tissue with various degrees of remodeling in the ischemic heart have not been fully understood. In this study, biophysical detailed single-cell models of ischemia 1a, 1b, and MI were developed to mimic the electrophysiological remodeling at different stages of ACS. 2D tissue models with different distributions of ischemia and MI areas were constructed to investigate the mechanisms of the initiation of reentrant waves during the progression of ischemia. Simulation results in 2D tissues showed that the vulnerable windows (VWs) in simultaneous presence of multiple ischemic conditions were associated with the dynamics of wave propagation in the tissues with each single pathological condition. In the tissue with multiple pathological conditions, reentrant waves were mainly induced by two different mechanisms: one is the heterogeneity along the excitation wavefront, especially the abrupt variation in conduction velocity (CV) across the border of ischemia 1b and MI, and the other is the decreased safe factor (SF) for conduction at the edge of the tissue in MI region which is attributed to the increased excitation threshold of MI region. Finally, the reentrant wave was observed in a 3D model with a scar reconstructed from MRI images of a MI patient. These comprehensive findings provide novel insights for understanding the arrhythmic risk during the progression of myocardial ischemia and highlight the importance of the multiple pathological stages in designing medical therapies for arrhythmias in ischemia.

## Author summary

Abnormal initiation or conduction of electrical impulses may lead to cardiac arrhythmias, which are very important cause of sudden and early death in developed countries. In many cases, cardiac arrhythmias are accompanied by ventricular fibrillation sustained by re-entry. The occurrence of reentrant arrhythmias is often associated with acute coronary syndrome, including three phases of myocardial ischemia 1a, 1b, and infarction. Previous studies have made lots of efforts to unravel the mechanisms of the initiation and maintenance of reentry waves during myocardial ischemia or infarction. However, the mechanisms of the initiation of reentrant waves in the tissue with multiple ischemic remodeling are not fully understood. Multi-scale computational models at the cell, tissue, and organ levels were developed in this study. The main finding in this study is that reentrant waves in the simultaneous presence of multiple ischemic conditions were mainly induced by the spatial heterogeneity of refractory periods caused by the co-action of action potential duration (APD) and CV along the wavefront, when the borders of different pathological conditions were perpendicular to the wavefront of the excitation wave. In addition, the increased excitation threshold of MI region as well as the impaired excitability of ischemia region can induce the generation of reentry, when the borders were parallel to the excitation wavefront. This provides insights into mechanisms of ventricular arrhythmias elicited by coexistence of multiple electrophysiological remodeling in ischemia.

## Introduction

ACS refers to a range of conditions related to suddenly diminished blood supply to the heart, including myocardial ischemia, infarction and so on [1]. During the progression of coronary artery stenosis or ACS, differential electrophysiological remodeling in ion currents was observed. For example, in the ischemia 1a stage (vascular obstruction <15min), I_Na_ decreased and activation delayed, I_NaL_ increased and I_CaL_ decreased, resulting in the impairment of the excitability of cell and the decrease of APD. In the ischemia 1b stage (vascular obstruction 15~45 min), I_CaL_ further decreased, and I_NaCa_ and I_NaK_ were also inhibited, resulting in the further decrease of APD. The long-lasting ischemia (vascular obstruction up to 3~5 days; referred as the MI stage in this study) produced the largest APD in these three phases due to the decline of I_Kr_ and I_Ks_, although I_Na_ and I_CaL_ were still inhibited to some extent in MI (see Table A and Figs A-B in S1 Text for details). At a certain stage, the simultaneous presence of multiple pathological conditions may exist, such as the coexistence of multiple electrophysiological remodeling in the case of heart failure [2]. In addition, the experimental data show that ischemia and myocardial infarction often coexist on the same tissue in the ischemic condition caused by coronary artery stenosis [3]. Previous studies have put numerous efforts to unravel the mechanisms responsible for the initiation and maintenance of reentry waves at both single-cell level and tissue level under the condition of single electrophysiological remodeling. For example, for the stage of ischemia 1a, Kazbanov *et al*. studied the effects of each single component (hyperkalemia, hypoxia, and acidosis) of ischemia on reentry wave propagation [4]. In addition, the inhomogeneous distribution of ion currents [5] and cross wall heterogeneity [6] in ischemia 1a will enhance the vulnerability to reentry. For the MI stage (i.e., the long-lasting ischemia), the changes of electrophysiological characteristics and the increased heterogeneous distribution of scar tissues will facilitate the generation and maintenance of reentrant waves [7,8]. However, the combined effect of multiple myocardial ischemic stages on the development of reentrant arrhythmias is rarely investigated.

In addition to the ionic remodeling, the spatial heterogeneity was shown as a key factor of inducing reentry waves in myocardial ischemia or MI. In 2D regional ischemic tissues [5], Romero *et al*. studied effects of spatial heterogeneity distribution of ion currents and ion concentration on reentry wave propagation, and showed that the mismatch of the source–sink relationship was the ultimate cause of the unidirectional block leading to reentry, rather than the dispersion of refractoriness. Weiss *et al*. studied the effects of spatial heterogeneity distribution of endocardial (Endo), intermediate layer (M), and epicardial (Epi) cells on reentrant wave propagation, and linked features of the ECG to ionic mechanisms in ischemic tissues [6]. Several studies on MI revealed the effect of the spatial heterogeneity distribution of scar areas on the occurrence of reentry waves [9–12]. However, most of these studies focused on the heterogeneity induced by Endo, M, and Epi cells, and the impacts of spatial heterogeneity induced by differential remodeling on the reentrant waves remain unclear.

In order to better understand the mechanisms of cardiac arrhythmias during the evolution of coronary artery blockage, multi-scale computational models at the cell, tissue, and organ levels based on the TP06 model (the ten Tusscher model published in 2006 [13]) were developed in this study. In 2D tissues, two different spatial distributions of electrophysiological remodeling were constructed, mimicking the distribution of multiple ischemic conditions within the 2D plane along and perpendicular to the direction of blood flow, respectively. In addition, the gradient distribution of ion currents and ion concentrations was taken into consideration in simulations of 2D tissues. In this study, corresponding to the two different distribution, two mechanisms underlying the initiation of reentry during ischemia were proposed, which are the spatial heterogeneity (especially the abrupt variation in CV across the border of different pathological conditions) along the excitation wavefront and the decreased SF for conduction at the edge of the tissue in MI region, respectively. Finally, the simulation results on the 3D ventricle tissue also demonstrated that the coexistence of multiple pathological conditions was more prone to induce reentry. The findings of this study sheds light on the arrhythmogenic mechanism during the progression of the coronary artery blockage, and is helpful to develop new therapeutic strategies for arrhythmias in myocardial ischemia and infarction.

## Methods

### Single cell models

Based on the experimental data, single cell models of ischemia 1a, 1b and MI were developed by incorporating the electrophysiological remodeling into the TP06 model [13,14]. Based on the physiological experimental data as shown in Table A in S1 Text, the conductance of ion currents, steady-state activation and inactivation curves, [ATP]_i_ and [K^+^]_o_ were modified to mimic remodeling in three pathological stages. In the ischemia 1a stage, the conductance of I_Na_, I_Ks_, I_CaL_ and I_to_ decreased, while the conductance of I_NaL_ increased. In addition, the steady-state activation curve of I_Na_ and both the steady-state activation and inactivation curves of I_to_ also were shifted. In the ischemia 1b stage, the conductance of I_NaK_, I_NaCa_, I_CaL_, I_rel_ and I_up_ decreased, while the conductance of I_bCa_ increased. In the MI stage, the conductance of I_Na_, I_CaL_, I_to_, I_Kr_ and I_Ks_ decreased. The details of parameter modification are shown in Table A in S1 Text. In our simulations, the value of intracellular adenosine triphosphate concentration ([ATP]_i_) was 6.8 mM in normal condition and decreased to 4.6 mM in ischemia [15] and MI [16] stages according to the experimental data. The effect of [ATP]_i_ variation in pathological conditions on cell action potential was integrated into the model via I_KATP_ whose formula was proposed by Clayton *et al*. [17]. Moreover, [K^+^]_o_ was set as 8mM during ischemia and MI according to the experimental data [11,12,16,18–25]. The experimental data of electrophysiological remodeling in different pathological conditions and the corresponding modifications of parameters in the computational models were summarized as shown in Table A in S1 Text in the supporting information.

The stimulation current was applied on the single cell with a stimulation strength of −86.2 pA/pF and a stimulus duration of 1 ms. The time step was set as 0.02 ms in our single cell simulation. A ventricular cell reached the steady state by 100 stimuli with a pacing duration of 1000 ms.

### 2D and 3D tissue models

The reaction-diffusion equation was used to model the mono-domain tissues [26]. The coupling between cells was isotropic in our simulations. The equation is modeled as follows:

$$\frac{\partial V_{m}}{\partial t}=\nabla \cdot D\nabla V_{m}-\frac{I_{ion}}{C_{m}}$$ (1)

where *V_m_* is the membrane potential, the value of the effective diffusion constant (D) is 0.154 *mm*^2^/*ms*, the value of the capacitance (*C_m_*) is 1*μF*/*cm*^2^, *I_ion_* is the total transmembrane current.

In addition to the remodeling in ionic currents, experimental data show that both ischemia1b [27–29] and MI [30] stages undergo a certain degree of cell-to-cell decoupling in the tissues (30~40%↓), giving rise to a reduction in the CV of excitation waves in tissues [29]. The remodeling in cell-to-cell decoupling was mimicked by reducing the diffusion coefficient (30%↓) in ischemia 1b and MI stages.

**Fig 1:**
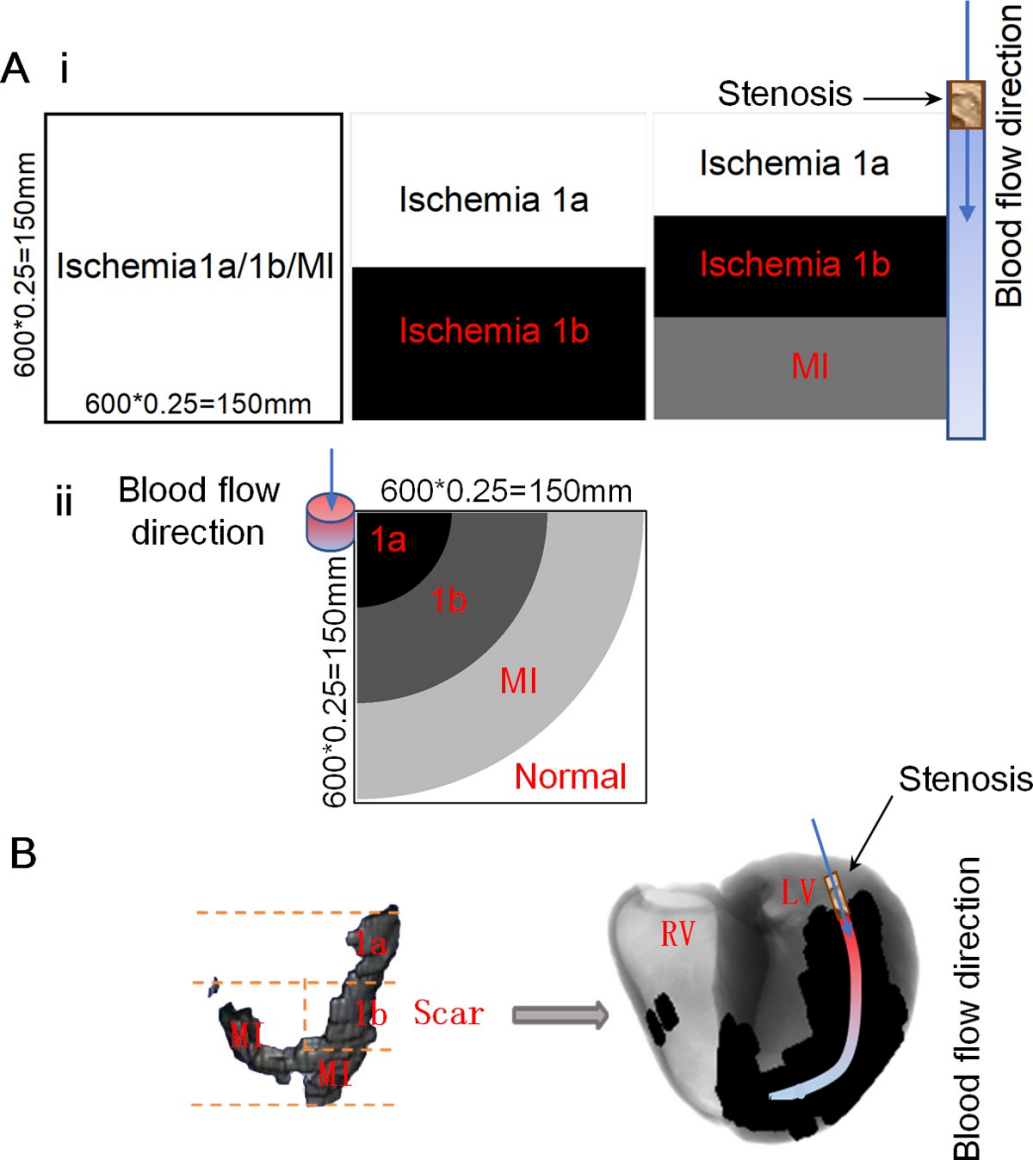
Schematic representation of the tissue models. (A) Tissue architecture of two different distributions of 2D ideal tissues with different pathological areas. (Ai) Distribution of multiple pathological conditions along the direction of blood vessel. The left, middle and right panels in (Ai) represent the tissue with only one pathological condition (left panel), with ischmia1a and 1b conditions (middle panel) and with ischmia1a,1b and MI conditions (right panel), respectively. (Aii) Distribution of multiple pathological conditions on the cross-sectional plane of the blood vessel. (B) Tissue architecture of the 3D real ventricle tissue with ischemia 1a, 1b, MI and normal areas.

The spatial distribution of various pathological conditions in 2D and 3D tissues was shown in Fig 1. In order to investigate the generation of reentry waves in the simultaneous presence of multiple ischemic conditions, two different spatial distributions of electrophysiological remodeling were constructed. One distribution is shown in Fig 1Ai (right panel) where ischemia 1a, 1b, and MI distributed horizontally, and excitation waves propagated vertically. In this case, the borders of various electrophysiological remodeling were perpendicular to the wavefront of excitation waves. On the contrary, as shown in Fig 1Aii, ischemia 1a, 1b, and MI distributed circularly in the other distribution. In this case when a stimulus was applied at the upper left corner, an excitation wave propagated circularly which is parallel to the borders of various electrophysiological remodeling. In this study, the structure distribution of ischemia 1a, 1b and MI areas in the same tissue designed in this paper is mainly based on the following idealized assumption: the farther away from the block site in the blood vessel (as shown in Fig 1), the higher the degree of ischemia. We assumed that the 2D ideal tissue in Fig 1Ai is supplied by only one vessel, and the direction of blood flow is top-down. When the vessel is occluded at the site marked the by arrow, the farther down from the occluded site, the higher the degree of ischemia will be. Therefore, the distribution structures of the three stages from top to bottom are corresponding to ischemia1a, ischemia1b, and MI, respectively, as shown in Fig 1Ai. In addition, for the 2D ideal tissue in Fig 1Aii, we assumed that the vessel located in upper left corner, and the blood flow is perpendicular to the panel of 2D tissue (as shown by the arrow in Fig 1Aii), therefore, ischemia 1a, 1b, MI and normal areas were a quarter of concentric circles from the upper left corner to the lower right corner areas.

For the 3D ventricular tissue model, 2D sections of the scar areas were extracted from the MRI images of a MI patient. These 2D slices were interpolated and reconstructed into a 3D scar which was then incorporated into the 3D human ventricle model according to our previous method [8]. Clinical studies have shown that obstruction of the anterior descending branch of the left coronary artery leads to ischemia of the anterior wall of the left ventricle and the interventricular septum [31,32], which is consistent with the scar areas of this patient. Similar to the assumption of the vascular blood supply in the 2D tissue simulations, the blood flow direction of the coronary artery is from top to bottom. Therefore, the reconstructed scar in the 3D ventricular tissue was equally divided into three parts (from top to bottom: ischemia 1a, ischemia 1b, and MI) as shown in Fig 1B. The tissues outside the scar areas are normal tissues. In addition, there may be multiple pathological tissues coexisting in circumferential direction due to collateral circulation in clinic. In this study, the span of pathological areas in the direction perpendicular to the blood flow is relatively narrow in comparison to the length along the blood flow from base to apex. Therefore, for simplicity, it is assumed that there are no multiple pathological areas in the direction perpendicular to the blood flow.

### Gradient distribution of all ion currents and ion concentration

In the previous section shown in Fig 1Ai, the region-wise heterogeneity caused sharp variations in electrophysiological characteristics at the borders, which was rarely seen in real heart. Therefore, it was assumed that the variations in electrophysiological characteristics across the tissue was gradually induced with the progress of myocardial ischemia, implicating a gradient-wise heterogeneity.

Decrease percentage of I_Na_ was set as gradient distribution from 11.3% (ischemia 1a) to 62% (MI). Increase percentage of I_NaL_ was set as gradient distribution from 150% (ischemia 1a) to 100% (MI). Decrease percentage of I_CaL_ was set as gradient distribution from 20% (ischemia 1a) to 50% (ischemia 1b) and from 50% (ischemia 1b) to 38% (MI). Decrease percentage of I_to_ was set as gradient distribution from 50% (ischemia 1a) to 63% (MI). [ATP]_i_ was set as gradient decrease from 6.8mM (ischemia 1a) to 4mM (MI). [K^+^]_o_ was set as gradient increase from 6mM (ischemia 1a) to 8mM (MI).

Decrease percentage of I_NaCa_ was set as gradient distribution from 100% (ischemia 1a) to 40% (ischemia 1b) and from 40% (ischemia 1b) to 100% (MI). Decrease percentage of I_NaK_ was set as gradient distribution from 100% (ischemia 1a) to 54% (ischemia 1b) and from 54% (ischemia 1b) to 100% (MI). Decrease percentage of I_Kr_ was set as gradient distribution from 100% (ischemia 1a) to 70% (MI). Decrease percentage of I_Kr_ was set as gradient distribution from 21.9% (ischemia 1a) to 80% (MI). Increase percentage of I_bCa_ was set as gradient distribution from 100% (ischemia 1a) to 130% (ischemia 1b) and from 130% (ischemia 1b) to 100% (MI). Decrease percentage of I_rel_ was set as gradient distribution from 100% (ischemia 1a) to 35% (ischemia 1b) and from 35% (ischemia 1b) to 100% (MI). Decrease percentage of I_up_ was set as gradient distribution from 100% (ischemia 1a) to 29% (ischemia 1b) and from 29% (ischemia 1b) to 100% (MI).

### The definition of SF

The calculation formula of SF improved by Romero *et al*. was used in this paper [33], as shown below:

$$SF=\frac{\int_{A}I_{c}dt+\int_{A}I_{out}dt}{\int_{A}I_{in}dt}A|t\in [t_{1\%dVm},t_{Vmax}]$$ (2)

$$I_{in}=\sqrt{I_{in,x}^{2}+I_{in,y}^{2}}$$

$$I_{out}=\sqrt{I_{out,x}^{2}+I_{out,y}^{2}}$$

where I_in_ is the axial current entering the cell, I_out_ is the axial current leaving the cell, I_c_ is the capacitive current and A is the integration interval, from the instant when membrane potential derivative reaches 1% of its maximum (t_1%dVm_) and to the instant of membrane potential peak (t_Vmax_) during the depolarization phase.

### Stimulation protocols of 2D and 3D tissue models

The detailed information of stimulation protocols was shown in the supporting information. The stimulation strength was -120 pA/pF in 2D and 3D simulations, and the stimulation duration was 3ms for 2D tissues and 2ms for 3D tissues. The 2D ideal tissues contained 600×600 nodes with a spatial resolution of 0.25 mm, resulting in a physical size of 150×150 mm^2^. In the 3D model, an isotropic domain composed of 325×325×425 nodes was used with a spatial resolution of 0.33 mm, corresponding to 107.25×107.25×140.25 mm^3^ in physical size. The standard S1-S2 protocol was used to initiate reentrant waves in the tissues and measure the VWs. For the S1-S2 protocol, five S1 stimuli with the interval of 1000 ms were applied on the leftmost three columns of nodes before S2 stimulus, which ensures the tissue reaching a steady state. S2 stimulus was applied in the upper left corner or lower left corner with the size of 300×300 cells. In the dynamic protocol, for the tissue in Fig 1Ai, stimuli were applied on the leftmost three columns of nodes, while, for the tissue in Fig 1Aii, stimuli were applied on a circular sector at the upper left corner with a radius of 5 nodes. In the 3D ventricular tissue, stimuli were applied on a small cubic tissue with the size of 10×10×10 nodes in intramyocardial regions.

### Implementation of the model

The TP06 cell model, written in C language, was downloaded from the website provided by ten Tusscher *et al*.. The differential equations were solved using the forward Euler method with a time step (Δt) of 0.02 ms. In 2D and 3D tissue models, the stability criterion and the Neumann boundary conditions were used [26]. An Intel core i7-3930K 64-bit CPU system was used to calculate the numerical solution, and parallel computing was applied for acceleration using a Gtx titan z GPU.

In addition, we’ve uploaded the code of this study to GitHub, with the following link: https://github.com/kodakfu/HeartNext/tree/main.

## Results

### Electrophysiological remodeling in single cell models

**Fig 2:**
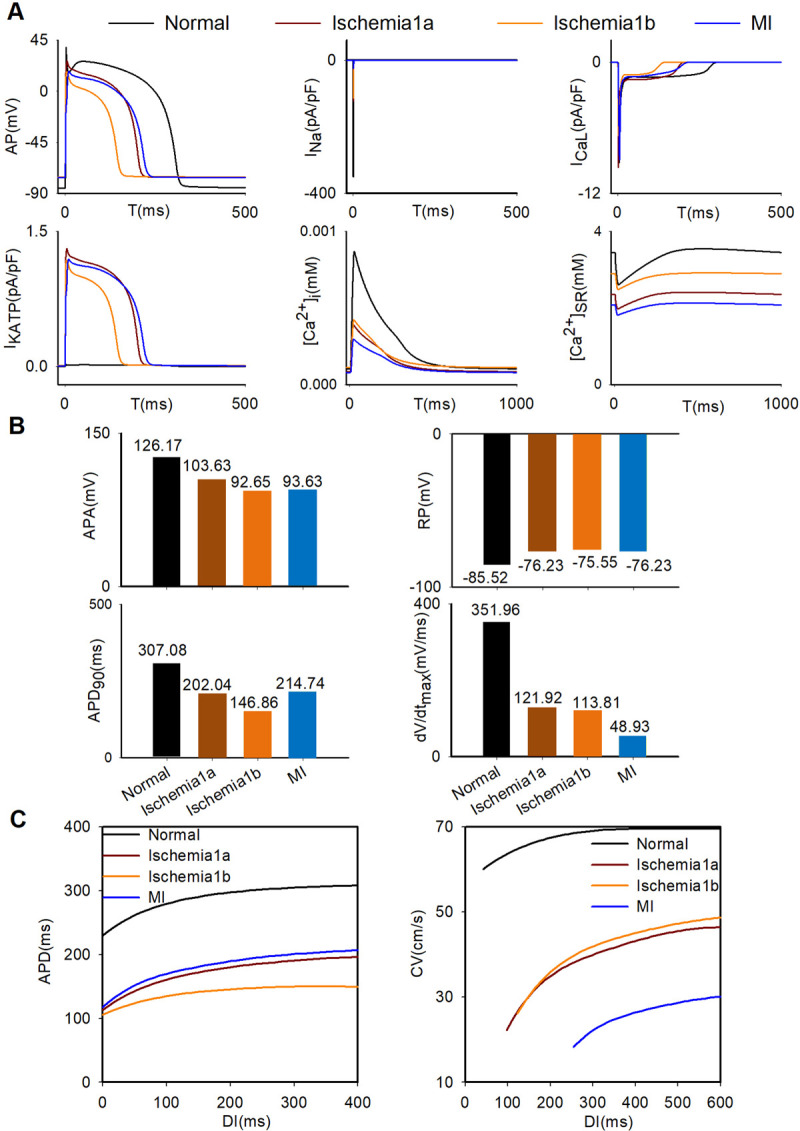
The variations of electrophysiological characteristics of single cells in normal, ischemia1a, ischemia1b, and MI. (A) Action potentials and several main ion currents in normal, ischemia1a, ischemia1b, and MI. (B) Values of APA, RP, APD_90_ and dV/dt_max_ in normal, ischemia1a, ischemia1b, and MI. (C) APD restitution curve using S1-S2 stimulation protocol in a single cell; CV restitution curve using dynamic stimulation protocol in a cable of 600 cells in four different conditions (normal, ischemia 1a, 1b and MI).

The single cell models of different pathological conditions were validated via comparing the variations of electrophysiological characteristics with experimental data as shown in Fig A in S1 Text in the supporting information. For the ischemia 1a stage, the APD was 202.04ms which is within the range of human experimental data (180-260ms) from Sutton *et al*. [34]. In addition, APA, RP and dV/dt_max_ are consistent with data of Dutta *et al*. [35]. For the ischemia phase 1b, the APD (146.86 ms) and APA (92.65 mV) were the smallest among these pathological stages, while the RP (-75.55 mV) was the highest. The APD in the ischemia 1b stage decreased by about half compared with that in the normal condition, which is basically consistent with the study of Pollard *et al*. [36]. This may result in a spatial heterogeneity of the electrophysiological characteristics during the progression from stenosis to occlusion in the coronary artery. In short, the simulation results of ischemia 1a and 1b are consistent with the previous studies [4,17,35,36]. For the MI stage, previous experimental data have shown that the APD is shortened [37–41]. However, it was shown that the APD was prolonged at the MI stage in many previous simulation studies [7,11,12,42,43]. Since it was reported that [ATP]_i_ was greatly decreased during the MI stage [16], in this study, this effect was incorporated into the model via I_KATP_ activation induced by the decrease of [ATP]_i,_ leading to a shortened APD in the MI stage. As shown in Fig 2B, compared with the APD (307.08 ms) in normal situation, the APD (214.74 ms) in the MI phase decreased significantly, which is consistent with the experimental data [39]. Also, the action potential amplitude (APA) (93.63 mV) and the resting potential (RP) (-76.23 mV) in the MI phase were agree with experimental data [37,39]. Notably, compared with the normal condition, the maximum depolarization rate (dV/dt_max_) (48.93 mV/ms) decreased significantly in the MI stage and was the smallest among three pathological conditions as shown in Fig 2B. In summary, the simulation results of electrophysiology remodeling in three pathological conditions were consistent with the experimental data, which validated the developed single models of ischemia 1a, 1b, and MI stages.

In addition, APD and CV restitution curves were shown in Fig 2C. The maximum slope value of APDR in normal condition (0.81) as shown in Fig C in S1 Text is close to the value (0.73) measured by ten Tusscher *et al*. [44]. The maximum slope values of these pathological conditions are less than 1, from the minimum 0.43 in ischemia 1b condition to the maximum 0.82 in MI condition as shown in Fig C in S1 Text. However, the maximum slope values of CVR in these pathological conditions are larger than that in normal condition, which is easier to promote reentry.

### Vulnerability of 2D tissues to reentrant waves

**Fig 3:**
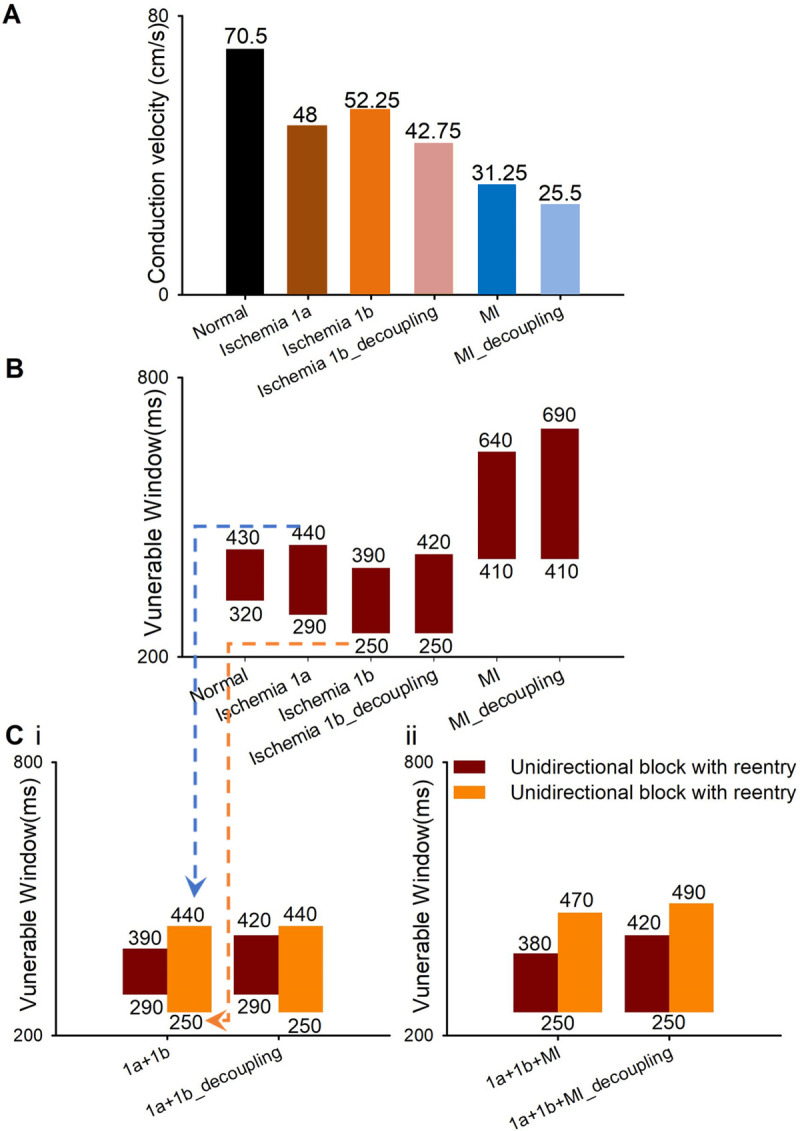
The CV and VWs of 2D ideal tissues in normal, single pathological and multiple pathological conditions (Fig 1Ai) using the S1-S2 protocol. (A) The CV in six different 2D homogenous tissues (Fig 1Ai, left panel): normal, ischemia 1a, 1b, MI, decoupled 1b and decoupled MI. (B) The VWs of 2D homogenous tissues (Fig 1Ai, left panel) when the S2 stimulus was applied in the lower left corner using the S1-S2 protocol. (C) The VWs of 2D tissues coexisting of multiple pathological conditions (i) where ischemia 1a and 1b distributed horizontally (Fig 1Ai, middle panel) and (ii) where ischemia 1a, 1b, and MI distributed horizontally (Fig 1Ai, right panel) when the S2 stimulus was applied in the (dark red) lower left or (orange) upper left corner using the S1-S2 protocol. (Notes: The dotted from Fig 3B to 3C represents that the upper (blue line) and lower (orange line) bound of the VM of the tissue with both ischemia 1a and 1b conditions are the upper bound of the VW of the tissue with ischemia 1a only and the lower bound of the VW of the tissue with ischemia 1b only, respectively).

VWs were measured in tissues with single and multiple pathological conditions to examine the effect of various remodeling on the vulnerability of cardiac tissue. In the tissues with a single pathological condition, the generation and maintenance of reentrant waves (Fig D in S1 Text) and the corresponding VWs (Fig 3B) were investigated using the S1-S2 cross-field protocol. The CV in each pathological condition decreased, comparing with that in normal conditions (Fig 3A), which is consistent with the relevant clinical trial records [29,45]. Among the ischemia1a, 1b, and MI tissues, the CV in MI tissue was the lowest (31.25 cm/s), while the CV in ischemia 1b tissue was the highest (52.25 cm/s) as shown in Fig 3A. In addition, inevitably, cell-to-cell decoupling further reduced the CVs of the tissue with ischemia 1b and MI to 42.75 cm/s and 25.5 cm/s, respectively. Meanwhile, compared with the VW of normal tissues (110 ms), the VWs in tissues with ischemia 1a, 1b, and MI alone increased to 150 ms, 140 ms, and 230 ms, respectively. Cell-to-cell decoupling further enlarged the VWs of the tissue with ischemia 1b and MI to 170 ms and 280 ms, respectively. These simulation results showed that the size of VWs is inversely proportional to the CV of the tissue, which is consistent with previous study [46].

Then, the reentrant waves and VWs were investigated using the S1-S2 cross-field protocol in the inhomogeneous tissues with multiple pathological conditions. In the tissue with two pathological conditions (ischemia1a and 1b/decoupled 1b), the S2 stimulus was applied in the upper or lower left corner, which is corresponding to ischemia 1a or 1b region, respectively (Fig 1Ai). When the S2 stimulus was applied in ischemia 1a regions (upper left corner), the upper bound of the VW of the tissue with multiple pathological conditions (440 ms) was the upper bound of the VW of the homogenous ischemia 1a tissue. Meanwhile the lower bound of the VW of the tissue with multiple pathological conditions (250 ms) was the lower bound of the VW of the homogenous ischemia 1b tissue (Fig 3Ci, orange bars). Intriguingly, on the contrary, when the stimulus was applied in ischemia 1b regions (lower left corner), the upper and lower bound of the VW of the inhomogeneous tissue was the upper bound of ischemia 1b tissue and the lower bound of the ischemia 1a tissue, respectively (Fig 3Ci, dark red bars). This hypothesis still held true when cell-to-cell decoupling occurred in the ischemia 1b region (Fig 3Ci). Therefore, the VW of multiple pathologies tissues is determined by the upper and lower bounds of VWs of tissues with a single pathological condition.

**Fig 4:**
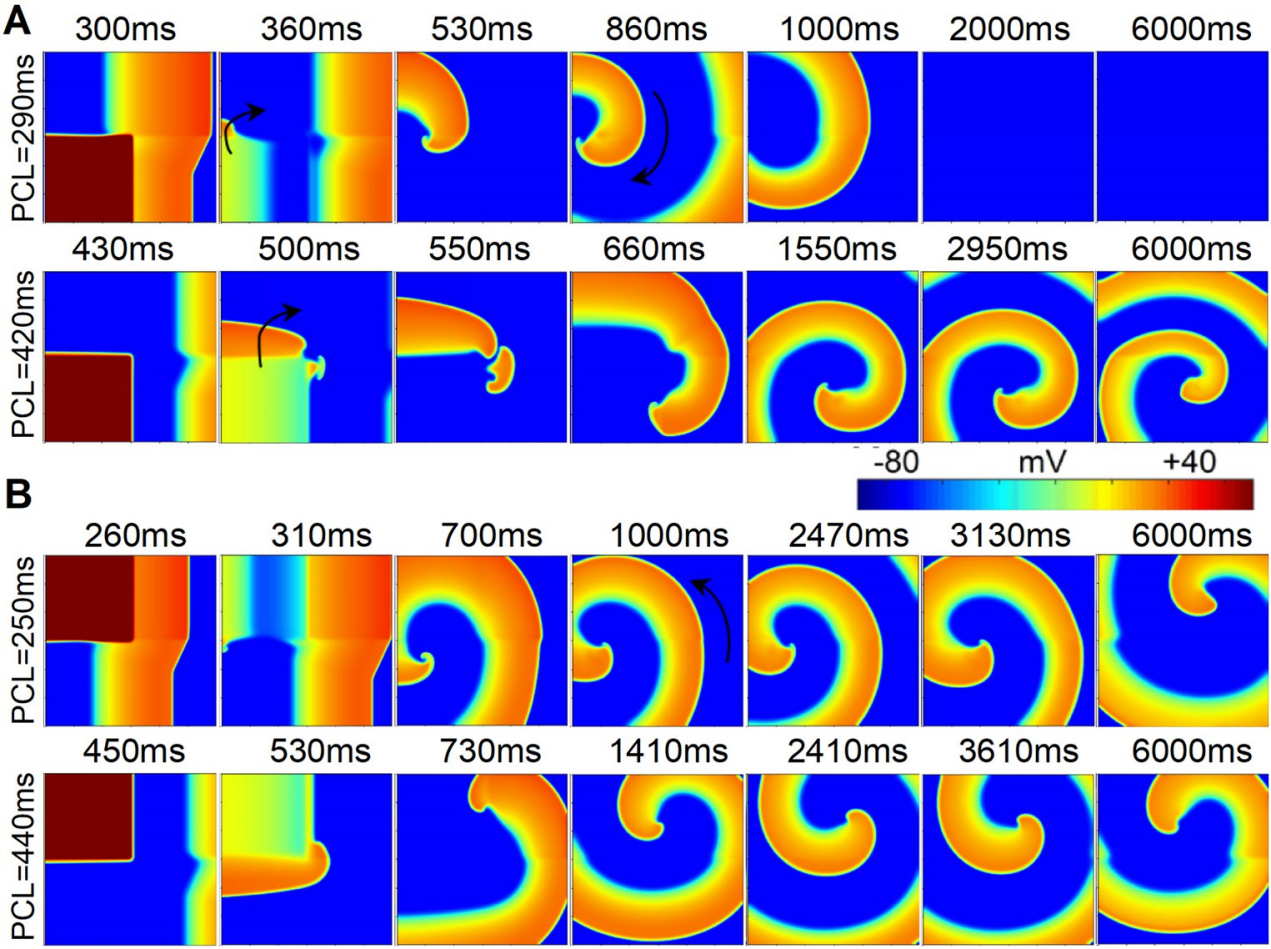
Wave propagation in the 2D tissue (Fig 1Ai, middle panel) where ischemia 1a and decoupled ischemia 1b distributed horizontally using the S1-S2 protocol when the S2 stimulus was applied in the (A) lower left or (B) upper left corner.

In the above hypothesis, when the S2 stimulus was applied in ischemia 1b regions (lower left corner, Fig 4A), the lower limit of the VW of the tissue with multiple pathological conditions is the limit allowing upward unidirectional conduction, i.e. the lower limit of the VW of the homogenous ischemia 1a tissue (Fig 4A, pacing cycle length (PCL) = 290ms). The upper limit of the VW of the tissue with multiple pathological conditions is the limit allowing bidirectional conduction of S2 stimulus, i.e. the upper limit of the VW of the homogenous ischemia 1b tissue (Fig 4A, PCL = 420ms). Similarly, when the S2 stimulus was applied in ischemia 1a regions (upper left corner, Fig 4B), the VW is determined by the limits of downward unidirectional conduction (Fig 4B, PCL = 250ms) and bidirectional conduction (Fig 4B, PCL = 440ms) of S2 stimulus.

In the tissue with three pathological conditions (ischemia1a, 1b, and MI), the upper and lower bounds of VWs were not exactly equal to the upper and lower bounds of the VW in the tissue with only one pathological condition (Fig 3Cii). The lower limit of the VW in the tissue with multiple pathological conditions is the time limit of S2 stimulus allowing upward/downwards unidirectional conduction (see white arrows in Fig E(A), 320ms and Fig E(B), 310ms at PCL = 250ms in S1 Text) when S2 was applied in the lower/upper corner. In this case, since the horizontal border of S2 region located within ischemia 1b region (see arrows in Fig E(A) and Fig E(B), 260ms at PCL = 250ms in S1 Text), the time limit of S2 stimulus allowing upward/downwards unidirectional conduction is determined by the electrophysiological properties of ischemia 1b tissue. Consequently, the lower limit of the VW in the tissue with multiple pathological conditions happens to be the lower limit of the VW in the homogenous ischemia 1b tissue (Fig 3Cii, 250ms). However, since the vertical border of S2 region located across multiple areas (ischemia 1a and 1b or ischemia1b and MI, see white arrows in Fig E(A), 480ms at PCL = 470ms and B, 450ms at PCL = 440ms in S1 Text), the upper limit of the VW (i.e., the limit allowing bidirectional conduction of S2 stimulus) is influenced by the excitation wave propagation across two regions (ischemia 1a and 1b or ischemia 1b and MI, Fig E in S1 Text). Although the upper limit of the tissue with three pathological conditions could not be precisely determined by any uniform tissue with ischemia 1a, 1b or MI, the VW of the tissue with three pathological conditions was larger than that in the tissue with ischemia 1a and 1b condition (Fig 3Ci and 3Cii).

### Initiation of reentrant waves in 2D tissues with multiple pathological conditions

**Fig 5:**
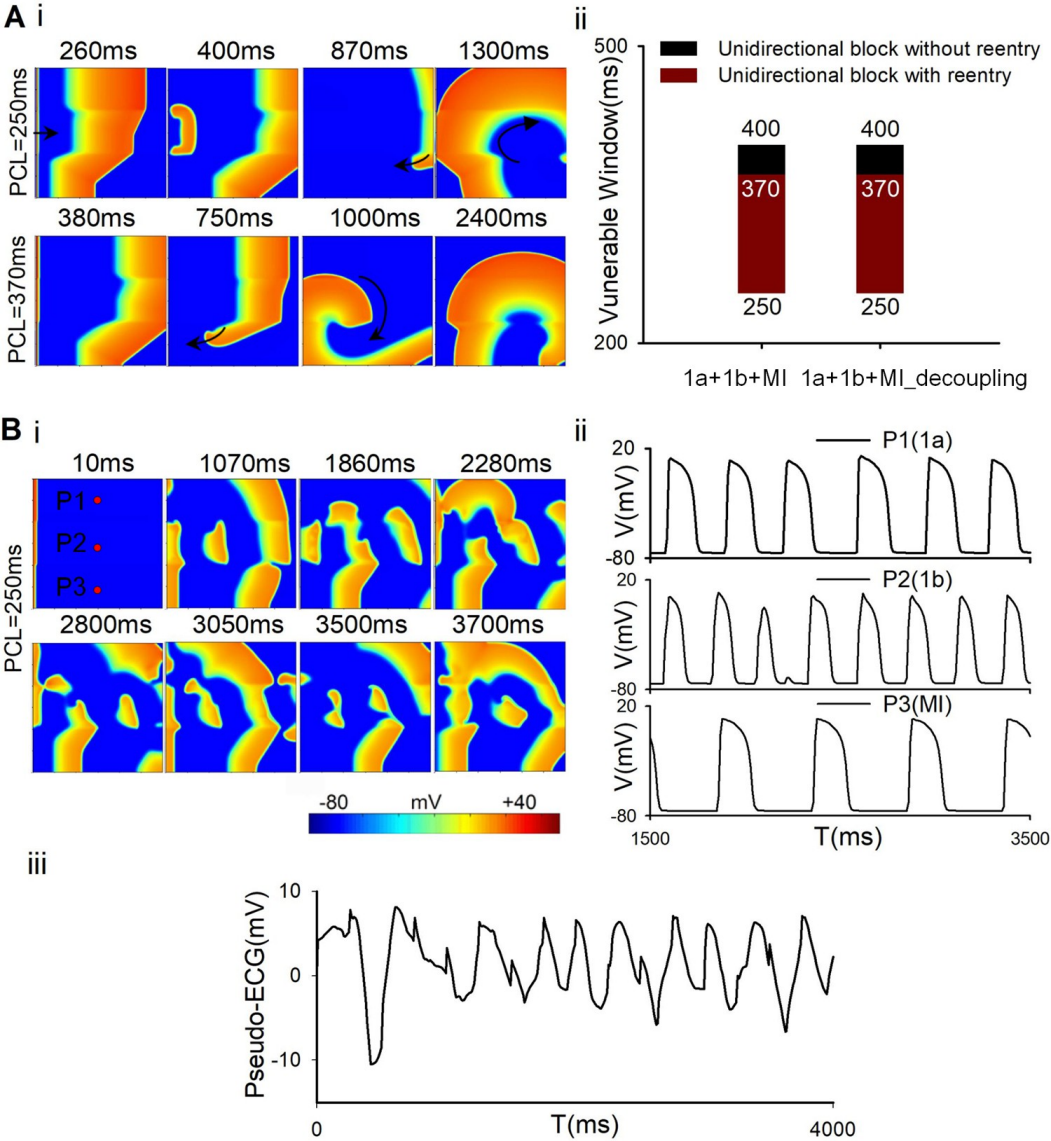
Wave propagation in the 2D tissue where ischemia 1a, decoupled 1b, and decoupled MI distributed horizontally (Fig 1Ai, right panel) when the leftmost stimulation was applied using S1-S2 protocol (A) or dynamic stimulus protocol (B) (see the supporting information for detailed protocols). (A) (i) Wave propagation in the 2D tissue and (ii) corresponding VWs when the leftmost stimulation was applied using S1-S2 protocol. (B) (i) Wave propagation, (ii) action potentials of points P1, P2 and P3 and (iii) Pseudo-ECG in the 2D tissue when the leftmost stimulation was applied using dynamic stimulus protocol.

In order to reveal the mechanisms underlying the genesis of reentry waves in 2D tissues, excitation wave propagation was investigated in the simultaneous presence of multiple ischemic conditions with two different distributions. Firstly, the reentrant waves were investigated when the spatial heterogeneity distribution was perpendicular to the excitation wavefront. In this section, both S1 and S2 stimuli were applied to the leftmost 3-line cells to induce reentrant waves (Fig 5). Different from the previous section, using this stimulation mode, reentrant waves were induced in multiple pathological tissues with ischemia 1a, 1b, and MI, but not in homogeneous 2D tissues. The VWs of the occurrence of reentrant waves were explored using the S1-S2 protocol (Fig 5A). In this case, the reentrant wave was induced at the border between ischemia 1b and MI regions due to the conduction block of S2 in MI area. The unidirectional conduction was accounted for the long refractory period of MI induced by the low CV in MI region (Fig 5Ai). Surprisingly, the size of VWs was the same in coupling and decoupling conditions (as shown in Fig 5Aii). This may be explained by the fact that in the cell-to-cell decoupling condition, although the CVs decreased in both ischemia 1b and MI regions, the variation of CV across their border remained unchanged, i.e., the decline in CV across the border was ~40% in coupling (52.25 to 31.25 cm/s, Fig 3A) and decoupling (42.75 to 25.5 cm/s, Fig 3A) conditions. When stimuli were applied to the leftmost 3-line cells using the dynamic stimulation protocol with a pacing cycle of 250ms (the detail protocol was shown in the supporting information), spiral wave breakup occurred in the 2D tissue (Fig 5Bi), leading to severely deformed action potentials at point P2 (Fig 5Bii) and thus an arrhythmic pseudo-ECG of the tissue (Fig 5Biii).

**Fig 6:**
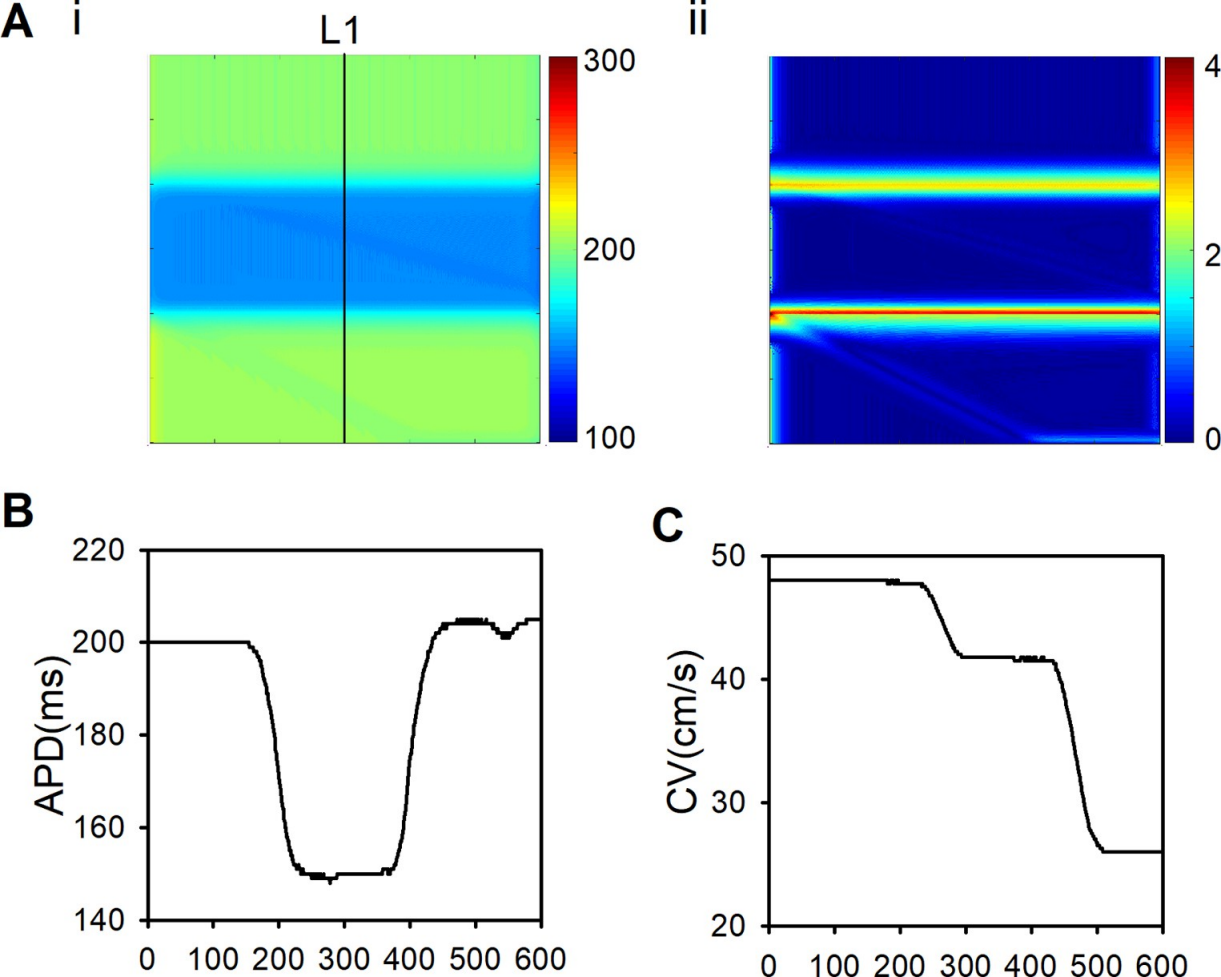
APD distribution, APD and CV of all cells along the line L1 of the first stimulation in the 2D tissue (Fig 1Ai, right panel) where ischemia 1a, decoupled 1b, and decoupled MI distributed horizontally when the leftmost stimulation was applied using S1-S2 protocol with a pacing cycle of 250ms. (A) (i) APD distribution of the first stimulation in the 2D tissue. (ii) The maximum APD difference between each cell and its neighbors in the 2D tissue. (B) APD of all cells along the line L1 in the 2D tissue. (C) CV of all cells along the line L1.

Fig 6A showed the spatial distribution of APD in the 2D tissue was perpendicular to the excitation wavefront which indicated by the line L1 in Fig 6Ai. It was shown that the largest APD gradient is located at the junction of different pathological tissues (Fig 6Aii). The difference of APD between ischemia 1a and 1b was similar to that between ischemia 1b and MI (Fig 6B), but the decline in CV between ischemia 1b and MI was much greater than that between ischemia 1a and 1b (Fig 6C). It suggested that multiple electrophysiological remodeling of various pathological conditions produced sharp heterogeneity along the excitation wavefront (the line L1). The formation of reentry waves is mainly due to the difference of CV between ischemia 1b and MI (see the details in Discussion).

**Fig 7:**
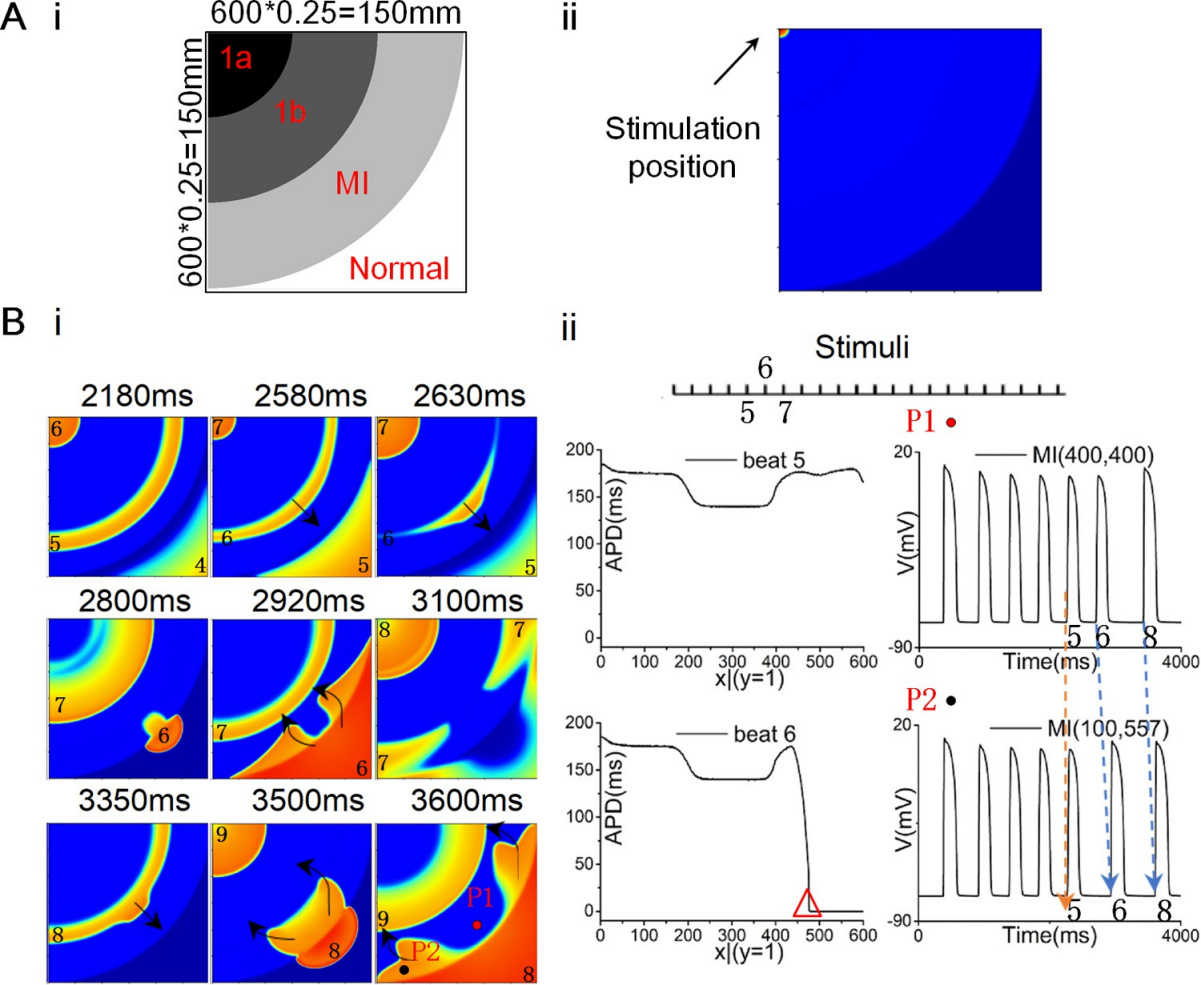
The 2D tissue structure from Fig 1Aii where ischemia 1a, decoupled ischemia 1b and decoupled MI distributed circularly, and wave propagation in the 2D tissue, when the upper left corner stimulation was applied using dynamic stimulation protocol with the stimulation interval of 420ms. (A) (i) The 2D tissue structure and (ii) the location of point stimulation. (B) (i) Wave propagation in the 2D tissue with ischemia 1a, decoupled 1b, and decoupled MI. (ii) APD of all cells along the border of the 2D tissue at the 5th and 6th stimuli and action potentials at points P1 and P2.

In the other spatial heterogeneity distribution, the borders of different pathological regions were parallel to the excitation wavefront (as shown in Fig 7A). In this case, excitation wave propagation in the 2D tissues with multiple pathological conditions was investigated using the dynamic stimulation protocol. Fig 7Bi showed the development of reentrant waves in the 2D tissue with ischemia 1a, decoupled 1b, and decoupled MI when the stimulation interval was 420ms. In this process, the excitation wave was blocked at marginal areas in MI region (Fig 7Bi, 2630 ms). With the propagation of the excitation waves along the diagonal line, the excitation site of the 6th stimulus located on the boundary between MI and normal areas (Fig 7Bi, 2800 ms) resulted in backward waves (as shown by the black arrows in Fig 7Bi, 2920 ms) and generated the reentrant waves. Then, the reentrant wave collided with the excitation wave of the 7th stimulus, hindering the continued wave propagation. This process repeated in the following stimuli (Fig 7Bi, 3350-3600ms).

**Fig 8:**
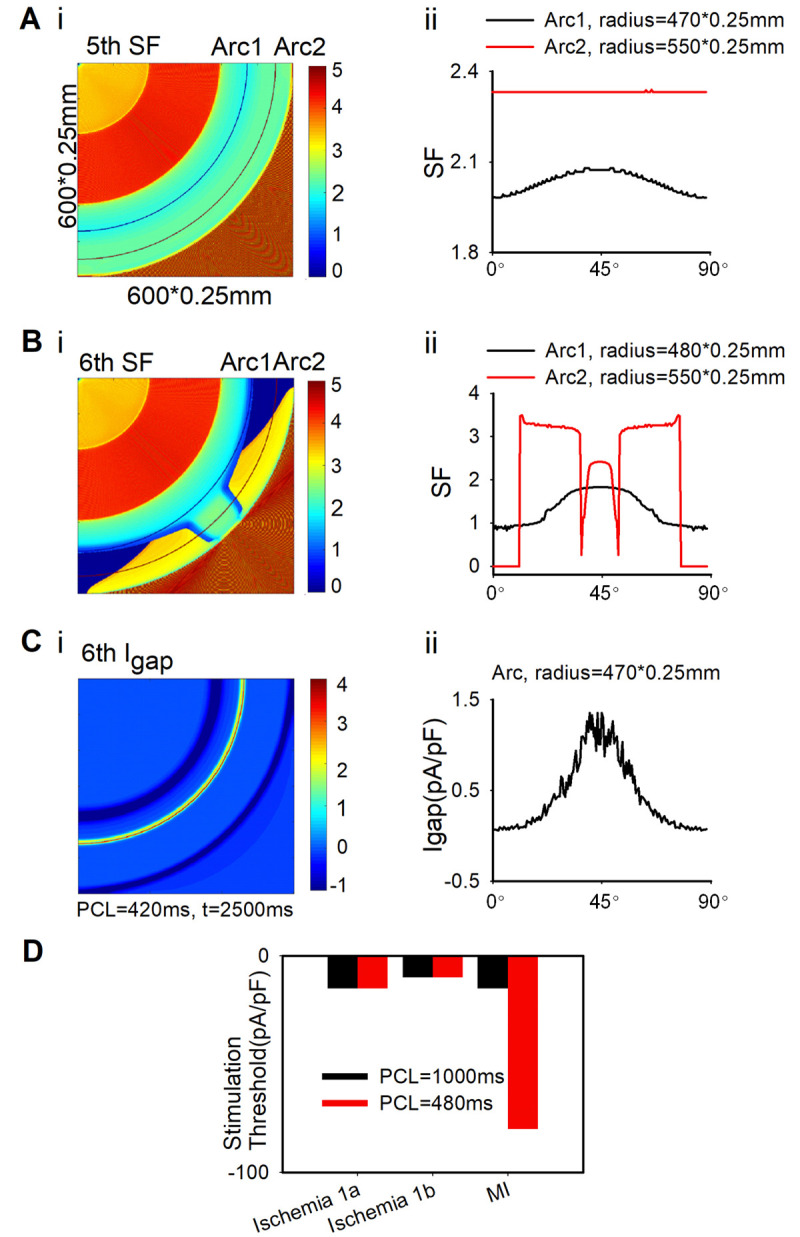
The distribution of SF and I_gap_ in the 2D tissue from Fig 1Aii where ischemia 1a, decoupled 1b, and decoupled MI distributed circularly using the dynamic stimulation protocol, where the point stimulation was applied on the upper left corner with the stimulation interval of 420ms. (A&B) The SF distribution in the 2D tissue at the (Ai) 5th and (Bi) 6th stimuli and (Aii and Bii) the SFs of all cells along Arc1 and Arc2. (C) (i) I_gap_ distribution in the 2D tissue and (ii) I_gap_ of all cells along the arc at 6th stimulation. (D) The minimum stimulation current for tissue excitability in the 2D tissue at different PCL (480ms; 1000ms) in ischemia 1a, 1b and MI conditions.

Different from the sharp heterogeneity along the excitation wavefront when the spatial heterogeneity distribution was perpendicular to the excitation wavefront (Fig 6), Fig F in S1 Text showed the spatial heterogeneity of APD along the excitation wavefront was trivial when the spatial heterogeneity distribution was parallel to the excitation wavefront. In order to reveal the mechanisms of the genesis of reentrant waves in this condition, the distribution of SF (see details in “The definition of SF” Methods) in the tissue was calculated. As shown in Fig 8, the SFs along Arc1(Fig 8A) were slightly smaller at the edge of the tissue (0° and 90°) in the 5th stimulation. This small variation in SF along the excitation wavefront gradually diminished (as shown in Arc 2, Fig 8A), and the excitation wave propagated normally in the 5th stimulation. However, in the 6th stimulation, the SFs at the edge of the tissue along Arc1(Fig 8B) further decreased to 0.875 (<1) and, therefore, led to the blockage of excitation waves at the edge (as shown in Fig 7Bi, 2630ms), which contributed to the backward excitation waves (Fig 8B) and induced reentry. The declined SFs at the edge areas mainly resulted from the decreased I_gap_ at the edge area along the excitation wavefront (Fig 8Cii).

### Reentrant waves in 3D tissue model

**Fig 9:**
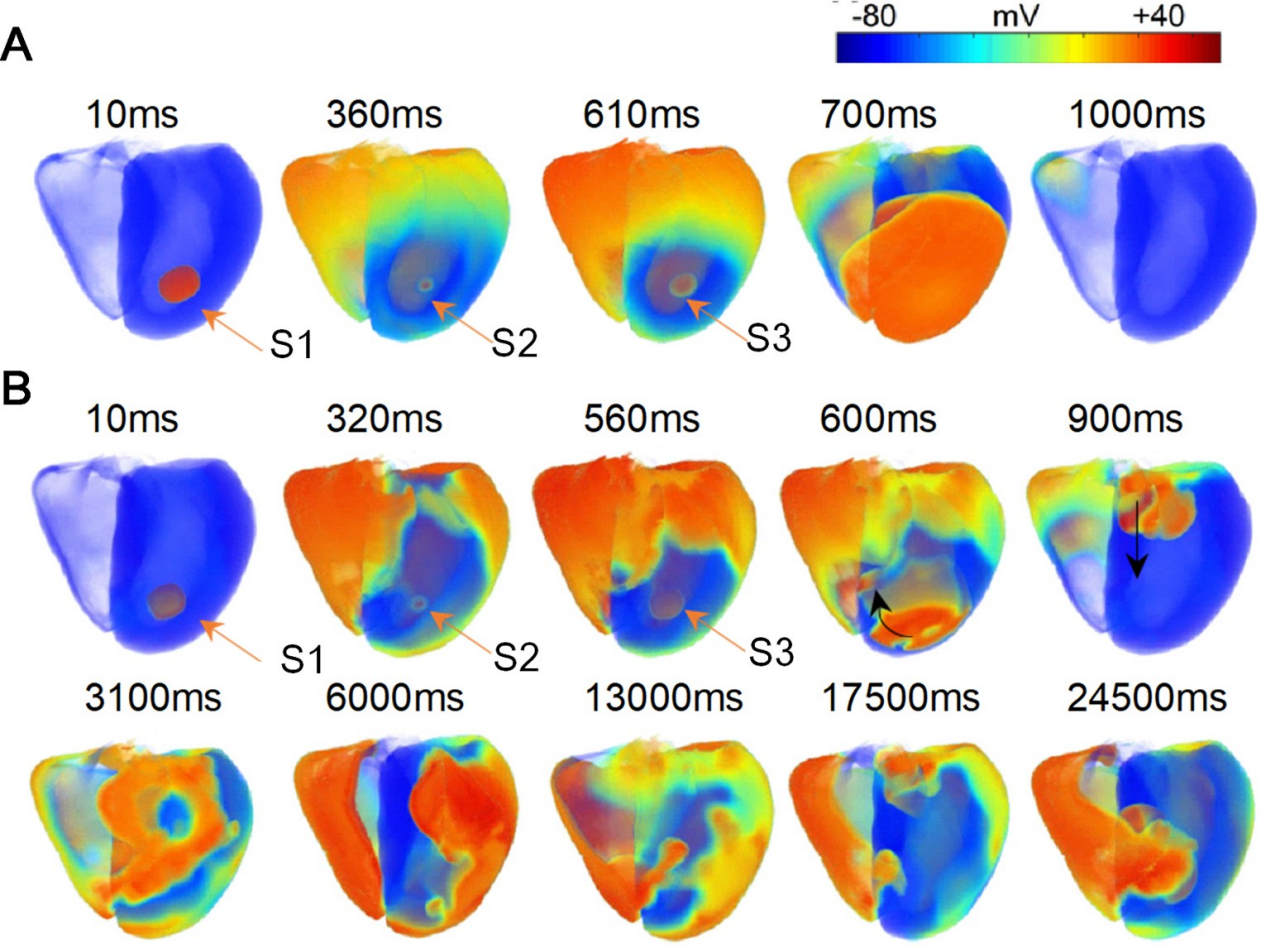
Wave propagation in the 3D ventricular tissue where three stimuli were applied on a small cubic tissue with the size of 10×10×10 nodes in intramyocardial region. (A) Wave propagation in the 3D ventricular tissue in normal conditions without MI scars. The S1, S2 and S3 stimuli were applied at 0ms, 350ms and 600ms, interval 350ms and 250ms, respectively. (B) Wave propagation in the 3D ventricular tissue with a scar containing ischemia 1a, 1b, and MI areas (see Fig 1B). The S1, S2 and S3 stimuli were applied at 0ms, 310ms and 550ms, interval 310ms and 240ms, respectively).

Fig 9A showed that in normal conditions, reentrant waves did not occur after S1, S2 and S3 stimuli. However, in the 3D ventricular tissue with multiple pathological conditions (Fig 1B), the slowed CVs in the pathological regions resulted in a slowed and heterogeneous repolarization (as shown in Fig 9B, 560ms). When the S3 stimulus was applied, the unidirectional blockage of wave propagation occurred (as shown in Fig 9B, 600ms), which in turn triggered reentrant waves (Fig 9B, 900ms). When the reentrant waves arrived the scar areas again, wavefront breakup was induced, giving rise to more disordered reentrant waves (Fig 9B, 1800ms). The reentrant waves persistently existed within 26000ms. Notably, using the same stimulation mode, reentrant waves were induced in the tissue with ischemia 1a only, but the duration of existence was relatively short (8000 ms) as shown in Fig G in S1 Text. Moreover, no reentry wave was induced in the tissue with ischemia 1b or MI only as shown in Fig G in S1 Text. It suggested that the coexistence of multiple pathological conditions in the tissue was more prone to cardiac arrhythmias.

## Discussion

With the development of ischemia, different electrophysiological remodeling occurred over time. Many studies have explored the effects of electrophysiological remodeling and the heterogeneous distribution of tissues on the wave propagation in the ischemia [5,47] and infarcted tissues [6,10]. However, the mechanisms of initiation of reentrant waves in the tissue with multiple ischemic remodeling are not fully understood. In this paper, the mechanism of cardiac arrhythmias caused by myocardial ischemia and MI during coronary artery occlusion was systematically investigated. The main findings of this studies are: (1) Computational models of ischemia 1a, 1b, and MI stages were developed and validated using experimental data. (2) The size of VWs was inversely proportional to the CV of the tissue. In 2D tissue, the VWs of tissues with multiple pathological conditions were determined by the VWs of tissues with a single pathological condition. In addition, in the tissue with three pathological conditions, the VW was larger than that in the tissue with ischemia 1a and 1b conditions, and could be further enlarged by cell-to-cell decoupling. (3) When the spatial heterogeneity distribution is perpendicular to the excitation wavefront, the formation of reentrant waves is caused by the spatial heterogeneity of refractory periods due to the interaction of APD and CV along the wavefront. And, the heterogeneity of the CV between ischemia 1b and MI on the wavefront plays a major role. (4) When the spatial heterogeneity distribution is parallel to the excitation wavefront, the elevated excitation threshold in MI region resulted in the decline of I_gap_ and the conduction block at the edge of the tissue along the excitation wavefront, producing the reentrant waves.

### Model development and model sensitivity

Many studies have focused on the electrophysiological variations at different stages during progression of ischemia in the last few decades, such as the studies of ischemia 1a (Kazbanov *et al*. [4], Clayton *et al*. [17], and Weiss *et al*. [6]), ischemia 1b (Alday *et al*. [48] and Pollard *et al*. [36]) or MI (Connolly *et al*. [7], Arevalo *et al*. [11], Deng *et al*. [12,42], and McDowell *et al*. [43]) individually. In this study, computational models of ischemia 1a, 1b and MI were developed based on the detailed experimental data of variations of electrophysiological characteristics (Table A in S1 Text). The APs generated in each pathological condition were consistent with previous studies (see Fig A in S1 Text). Especially, for the stage of MI, although the experimental data show that APD is shortened [37–41], APDs produced by previous computational models are prolonged [7,11,12,42,43]. In this study, a transmembrane current of I_KATP_ [16] was incorporated into the single cell of MI. The activation of I_KATP_ due to the decreased [ATP]_i_ resulted in a shortened APD in MI, which is consistent with the physiological experimental data [16].

In order to investigate the sensitivity of single cell models, each parameter shown in Table A in S1 Text was increased and decreased by 10% (individually and collectively) to simulate the impacts of different parameters on the action potentials in ischemia 1a, 1b and MI stages (Figs H-J in S1 Text). It was demonstrated that changes of I_CaL_ and I_KATP_ are the main factors of the APD variations, and the change of [K^+^]_o_ is associated with the variations in RPs for all three stages. The remodeling of other currents during ischemia has a minor effect on the action potential.

In 2D simulations, previous studies have focused on the effects of spatial heterogeneity of a single pathological condition (either transmural [6] or gradient heterogeneity [5,47]) on wave propagation. In this study, the different spatial distributions of multiple ischemic stages were investigated, providing insights into the risk of cardiac arrhythmias presented during the progression of ischemia.

### The properties of the VW of tissues coexisting of multiple pathological conditions

In the tissue with multiple pathological conditions, the VW was different when the S2 stimulus was applied at lower or upper left corner (Fig 3C). The upper and lower bounds of the VW were determined by the time limits of bidirectional (horizontal) and unidirectional (vertical) propagation of S2 stimulus, respectively. In the tissue with two pathological conditions (i.e. ischemia 1a and 1b, Fig 4), the upper bound of the VW happened to be the upper bound of the VW measured in a uniform tissue with one of the pathological condition, while the lower bound was coincidentally the lower bound measured in a uniform tissue with the other pathological condition. However, when a tissue contains more than two pathological conditions, the bounds of VW were influenced by dynamics of wave propagation in the tissues with each single pathological condition and cannot be obtained directly. What’s worse, the heterogeneous distribution of pathological regions in real patient makes the situation more complicated. Therefore, a new parameter should be developed to quantify the risk of reentry in the future studies with multiple pathological conditions. In this study, the VW of the tissue with three pathological conditions was larger than that in the tissue with ischemia 1a and 1b conditions (Fig 3C), suggesting a higher risk of arrhythmias.

### The mechanisms of reentry initiation with different spatial distributions of multiple pathological conditions

In this study, two spatial distributions of multiple pathological areas were designed to investigate the excitation wave propagation within the 2D plane along and perpendicular to the direction of blood flow. When the spatial heterogeneity distribution is perpendicular to the excitation wavefront (i.e., within the 2D plane along the direction of blood flow, Fig 1Ai), reentry was not observed in the tissue with both ischemia 1a and 1b, but was seen in the tissue with ischemia 1a, 1b, and MI (as shown in Fig 5), implicating that the excitation wave propagation across the border of ischmia1b and MI played a major role in initiating of reentry. In addition, the VW significantly declined when ischemia 1b area was replaced with ischemia 1a area (as shown in Fig K in S1 Text). At the border between different pathological conditions along excitation wavefront, sharp variations in APD and CV were produced (Fig 6). As the excitation wave length (WL) is the product of APD and CV, the excitability and refractoriness of the tissue across the border were altered significantly. At the border between ischemia 1a and 1b, the shortened WL and the declined CV counterbalanced with each other, leading to a non-significant difference between wave tails in ischemia 1a and 1b region (Fig 5Ai, 260ms). However, at the border between ischemia 1b and MI, the wave tail of MI region was significantly delayed (Fig 5Ai, 260ms) due to the sharp decline in CV (Fig 6C). The delayed wave propagation increased the refractory period in MI region, which was responsible for the initiation of reentrant waves. Therefore, the heterogeneity along the excitation wavefront (especially, the difference of CV between ischemia 1b and MI) was the main reason of reentry when the spatial heterogeneity distribution is perpendicular to the excitation wavefront.

When the spatial heterogeneity distribution is parallel to the excitation wavefront (i.e., within the 2D plane perpendicular to the direction of blood flow, Fig 1Aii), there was no spatial heterogeneity along the excitation wavefront at the beginning of stimulation. Since less cells were coupled at the edge of the tissue, I_gap_ was smaller (Fig 8C), leading to a weaker drive force for excitation. Moreover, the excitation threshold in MI region was dramatically increased at high pacing frequency (Fig 8D), implicating that the drive force required for excitation in MI region was enlarged. In this case, the SF for conduction at the edge in MI region was gradually decreased (Fig 8A and 8B), which eventually gave rise to the conduction block at the edge and promote the formation of reentrant waves (Fig 7). What’s more, if the ischemia 1b area was replaced with the ischemia 1a area, the VW declined from 50 ms to 40 ms. Compared with the ischemia 1a stage, the lower APA, dV/dt_max_ and CV as well as the higher RP impaired the excitability of ischemia 1b region and thus impeded the wave propagation, suggesting an important role of ischemic 1b in initiation of reentry. As the edge of ischemic region in real heart tissue is much more complicated than the ideal tissue in this study, the risk of reentrant arrhythmia would be amplified when multiple pathological conditions coexisted.

### Initiation of reentrant waves in the tissue with gradient heterogeneity

**Fig 10:**
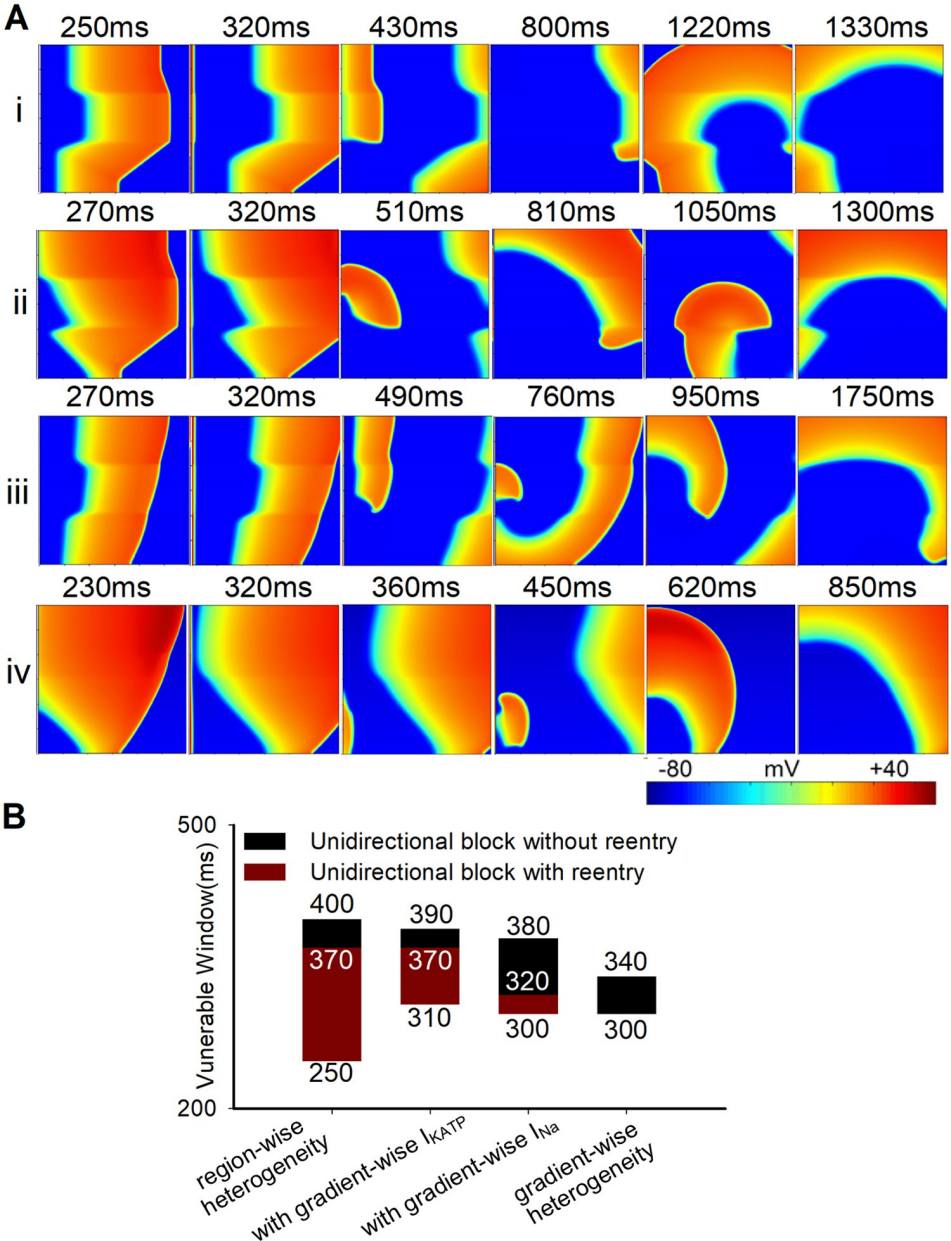
Wave propagation and the VWs in four different conditions in the 2D tissue with ischemia 1a, 1b, and MI when the leftmost stimulation was applied using S1-S2 stimulation protocol. (A) Wave propagation in the 2D tissue in four different conditions with the S2 stimulation interval of 310ms. (i) Region-wise heterogeneity, (ii) region-wise heterogeneity with gradient-wise I_KATP_, (iii) region-wise heterogeneity with gradient-wise I_Na_, (iv) gradient-wise heterogeneity for all parameters (see details in Methods). (B) The distribution of VWs in the 2D tissue in four different conditions.

During the progression of ischemia in the real heart, sharp borders of different pathological regions were rarely seen. Therefore, the gradient distributions of several parameters were set across the 2D tissue to investigate the mechanisms underlying reentry (the details about the design of gradient distribution were shown in the part of the method). Reduced VWs of reentrant waves were observed when I_KATP_ and I_Na_ were set as gradient distribution individually (Fig 10), as the gradient-wise heterogeneity of I_KATP_ or I_Na_ corresponded to a decreased heterogeneity of APD or CV along the wavefront, respectively. However, the gradient distribution of I_Na_ had a more profound effect in reducing the VW, suggesting that the heterogeneity of the CV, rather than the APD, was the main factor of the genesis of reentry, which was consistent with the conclusion in region-wise heterogeneity. Although reentry was not induced when all the remodeled parameters were set as gradient distribution, breakup of reentry waves was induced using the dynamic stimulation protocol (as shown in Fig L in S1 Text), indicating the potential risk of arrhythmia.

Although the gradient distribution may be more common in clinical scenarios, when certain pathological conditions suddenly occur (e.g. acute coronary occlusion), there would produce a sharp change in electrophysiological parameters at the boundary, which is corresponding to the non-gradient distribution and would be proarrhythmogenic.

At present, simulation researches related to ischemia only show that the occurrence of arrhythmias is closely related to the dispersion of spatial refractory period, but there is no detailed discussion on the mechanism of arrhythmias under different wave propagation conditions. Our study showed that the mechanisms of arrhythmias were different when the direction of wave advance was perpendicular or parallel to the distribution of spatial heterogeneity. In clinical practice, the direction of excitation wave propagation is generally between perpendicular and parallel to the spatial heterogeneity distribution. This paper will further improve the study on the mechanism of arrhythmias induced by ischemia, and could bring some inspiration for the study of other multiple pathological stages.

### Clinical implications

This study revealed the risk and the mechanism of arrhythmias in the presence of multiple pathological conditions, and found that the presence of ischemia 1b significantly increased the risk of ventricular arrhythmias. Therefore, for a patient with myocardial ischemia, a clinical care team should pay more attention to time window of ischemia 1b generation (15–45 min) and the following stage with possible multiple pathological conditions. In addition, for the treatment, mapping and ablation attempt to identify the crucial isthmus where the excitation wave conduction is slow [49]. The border between MI and other pathological areas, especially at the edge of the tissue, may be a curial target for radiofrequency ablation as it is more likely to induce reentry in this region while more complex spatial dispersion of repolarization presents in clinics.

### Limitation

The limitations of this paper exist in the following aspects. At single cell-level, the limitations of TP06 cell model also exist in our simulation, for example, the effect of CaMKII is not included. In addition, it depends on the further development of patch-clamp experimental studies of human cells in ischemia 1a, 1b, and MI, so that more real human data can be used to remodel single cells. In addition, the effect of the heterogeneity of the three cardiomyocyte cells (Endo, M, Epi cells) was not compared in our single-cell and 2D simulation experiments, only the Epi cells were used. However, this does not change the conclusions in single-cell and 2D tissue simulations, and these three different cells were included in our 3D real ventricular tissues. The stage of MI is often accompanied by fibrosis, while fibrosis tissues were not added into our models, which can be supplemented in the subsequent simulation work. Furthermore, more diversified and complex distribution of ischemia 1a, 1b, and MI areas may be designed in 2D tissues and 3D tissues, but this will not change our conclusions. What’s more, to refine the division of pathological areas in 3D geometry, the center line of the blood vessel should be calculated properly to estimate the distance of each point to the source of blood supply, which remains a challenge and requires massive efforts. As the distribution of pathological areas in multiple direction in 3D geometry is a crucial for arrhythmias, we will investigate this effect in details in our future studies. Due to the lack of 3D fiber orientation data at present, we did not consider the anisotropic diffusion in the process of 3D simulation. In addition, our current model does not take into account individual differences of patients, e.g. in 3D ventricular geometry. Individual differences between patients are a curial factor towards discerning individual risk and potentially revealing mechanisms while applying the current results in further research. Moreover, in the gradient distribution model, parameters were discretized in a relatively large parameter space. In this case, some parameters may be out of the scope of the normal physiological state, which should be paid attention to while interpreting these results. However, these results help to understand the dynamic behavior of the system in a large parameter space.

## Supporting information

S1 Text Supporting information: A: Simulation parameters and results in single cells, B: Stimulation protocols, C: Supplementary simulation results. Table A: The electrophysiological properties of the three stages. Fig A: Validation of single cell models in three stages of ischemia 1a, 1b and MI. Fig B: The variations of electrophysiological characteristics of other ion currents and concentration of single cells in normal, ischemia1a, ischemia1b, and MI conditions. Fig C: Maximum slopes of APD and CV restitution curves in four conditions (normal, ischemia 1a, 1b and MI). Fig D: Wave propagation in 2D homogenous tissues: normal, ischemia 1a, ischemia 1b, MI, decoupled 1b and MI. Fig E: Wave propagation in the 2D tissue where ischemia 1a, decoupled ischemia 1b, and decoupled MI distributed horizontally (Fig 1Ai, right panel) using the S1-S2 protocol when the S2 stimulus was applied in the (A) lower left or (B) upper left corner. Fig F: APD distribution and APD of all cells along lines L1, L2 and L3 of the fifth stimulation in the 2D tissue where ischemia 1a, decoupled 1b, and decoupled MI distributed circularly (Fig 1Aii), when the upper left corner stimulation was applied using dynamic stimulation protocol with a pacing cycle of 420ms. Fig G: Wave propagation in the 3D ventricular tissue (A) with ischemia 1a, (B) with ischemia 1b, or (C) with MI areas. Fig H: The change of cellular AP when each parameter changes alone and simultaneously in the single-cell model of ischemia 1a. Fig I: The change of cellular AP when each parameter changes alone and simultaneously in the single-cell model of ischemia 1b. Fig J: The change of cellular AP when each parameter changes alone and simultaneously in the single-cell model of MI. Fig K: Wave propagation in the 2D tissue where ischemia 1a, decoupled 1b, and decoupled MI distributed horizontally (Fig 1Ai, right panel) before and after ischemia 1b area was replaced with ischemia 1a area, when the leftmost stimulation was applied using the S1-S2 protocol. Fig L: Wave propagation in the 2D tissue where ischemia 1a, 1b, and MI distributed horizontally (Fig 1Ai, right panel) with gradient distribution of all parameters and action potentials of points P1, P2, and P3 when the leftmost stimulation was applied with a pacing cycle of 290ms using the dynamic stimulation protocol. Fig M: Tissue architecture of two new 2D ideal tissues, wave propagation and the distribution of VWs in the 2D tissue with ischemia 1a, 1b, and MI when the leftmost stimulation was applied in four different conditions using S1-S2 protocol.
(DOCX)
