# Supplementary material for: Mechanisms of ventricular arrhythmias elicited by coexistence of multiple electrophysiological remodeling in ischemia: A simulation study
Source: PLoS Comput Biol. 2022 Apr 27;18(4):e1009388. doi: 10.1371/journal.pcbi.1009388 (PMC9045648; doi:10.1371/journal.pcbi.1009388)
Supplement: S1 Text — Supporting information: A: Simulation parameters and results in single cells, B: Stimulation protocols, C: Supplementary simulation results. Table A: The electrophysiological properties of the three stages. Fig A: Validation of single cell models in three stages of ischemia 1a, 1b and MI. Fig B: The variations of electrophysiological characteristics of other ion currents and concentration of single cells in normal, ischemia1a, ischemia1b, and MI conditions. Fig C: Maximum slopes of APD and CV restitution curves in four conditions (normal, ischemia 1a, 1b and MI). Fig D: Wave propagation in 2D homogenous tissues: normal, ischemia 1a, ischemia 1b, MI, decoupled 1b and MI. Fig E: Wave propagation in the 2D tissue where ischemia 1a, decoupled ischemia 1b, and decoupled MI distributed horizontally (Fig 1Ai, right panel) using the S1-S2 protocol when the S2 stimulus was applied in the (A) lower left or (B) upper left corner. Fig F: APD distribution and APD of all cells along lines L1, L2 and L3 of the fifth stimulation in the 2D tissue where ischemia 1a, decoupled 1b, and decoupled MI distributed circularly (Fig 1Aii), when the upper left corner stimulation was applied using dynamic stimulation protocol with a pacing cycle of 420ms. Fig G: Wave propagation in the 3D ventricular tissue (A) with ischemia 1a, (B) with ischemia 1b, or (C) with MI areas. Fig H: The change of cellular AP when each parameter changes alone and simultaneously in the single-cell model of ischemia 1a. Fig I: The change of cellular AP when each parameter changes alone and simultaneously in the single-cell model of ischemia 1b. Fig J: The change of cellular AP when each parameter changes alone and simultaneously in the single-cell model of MI. Fig K: Wave propagation in the 2D tissue where ischemia 1a, decoupled 1b, and decoupled MI distributed horizontally (Fig 1Ai, right panel) before and after ischemia 1b area was replaced with ischemia 1a area, when the leftmost stimulation was applied using the S [file pcbi.1009388.s001.docx]

Mechanisms of ventricular arrhythmias elicited by coexistence of multiple electrophysiological remodeling in ischemia: a simulation study

Cuiping Liang^1^, Qince Li^1,2*^, Kuanquan Wang^1^, Yimei Du^5^, Wei Wang^1^, Henggui Zhang^2,3,4^

^1^ School of Computer Science and Technology, Harbin Institute of Technology (HIT), Harbin 150001, China.

^2^ Peng Cheng Laboratory, Shenzhen 518055, China.

^3^ School of Physics and Astronomy, The University of Manchester, Manchester M13 9PL, UK.

^4^Key Laboratory of Medical Electrophysiology of Ministry of Education and Medical Electrophysiological Key Laboratory of Sichuan Province, Institute of Cardiovascular Research, Southwest Medical University, Luzhou, China.

^5^Wuhan Union Hospital, Tongji Medical College of Huazhong University of Science and Technology, Wuhan, China.

* Corresponding author: qinceli@hit.edu.cn

**Supporting Information**

**A Simulation parameters and results in single cells**

Previous studies have shown that the action potential of cells during ischemia changed greatly compared with that in normal condition: the elevated RPs (caused by hyperkalemia[1-6]), the shortened APD (caused by the increase of I_KATP_[7, 8]), the decreased APAs, and the reduced dV/dt_max_ (caused by the decrease of I_Na_[9]), implying an impaired excitability in these conditions, providing the substrate for arrhythmias.


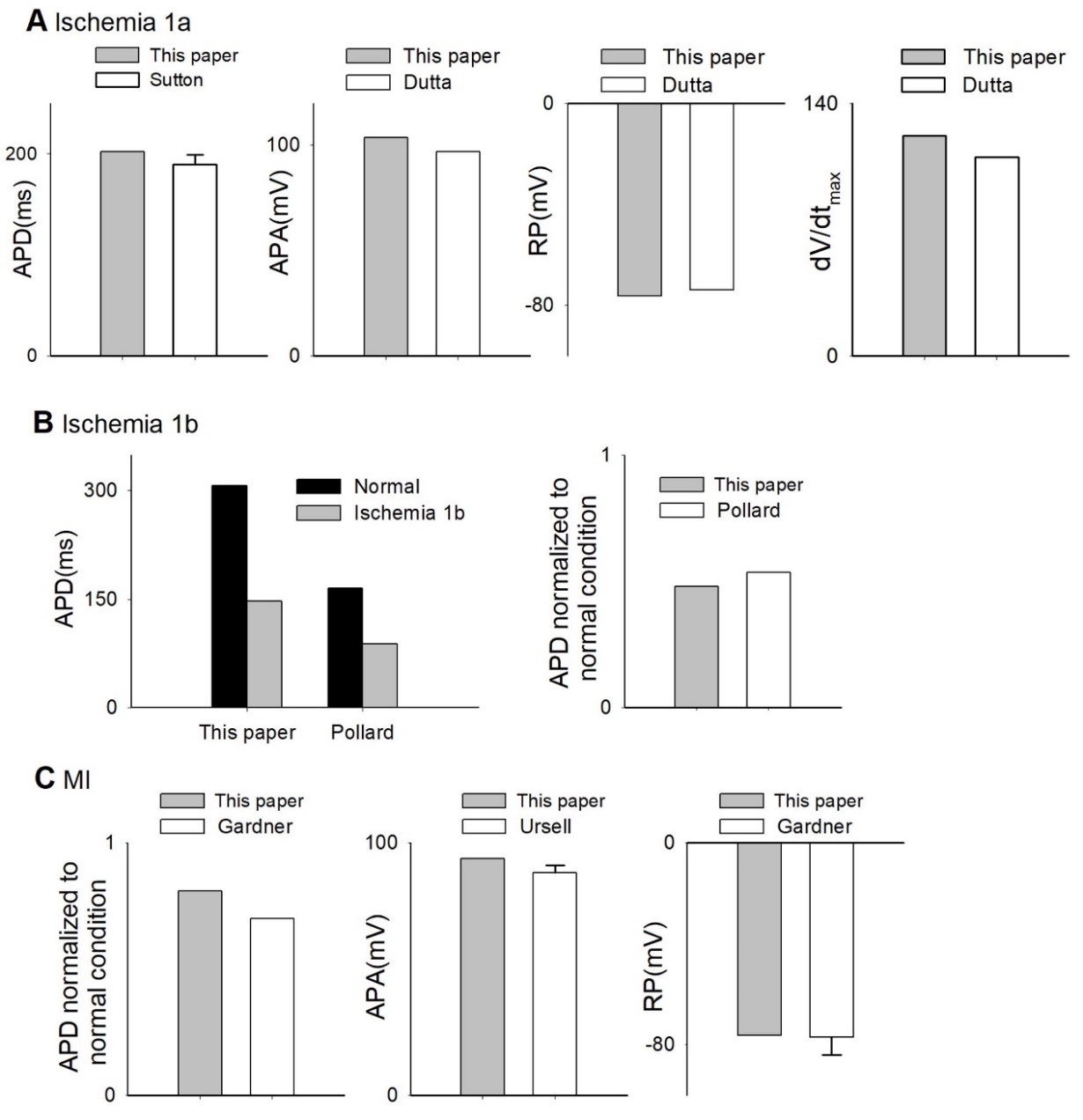


**Fig A.** Validation of single cell models in three stages of ischemia 1a, 1b and MI. (A) Validation of the single cell model in ischemia 1a with experimental data of Sutton *et al.*[10] and Dutta *et al.*[11]. (B) Validation of the single cell model in ischemia 1b with experimental data of Pollard *et al.*[12]. (C) Validation of the single cell model in MI with experimental data of Gardner *et al.*[13] and Ursell *et al.*[14].

| Table A. The electrophysiological properties of the three stages. | **Study** | 1[9]; | 1[15]; 2[16]; | 1[17]; | 1[18];2[19];3[20];4[21];5[22, 23]; | 1[23, 24]; 2[25]; 3[26]; 4[27-29] | 1[1]; 2[2, 28, 29];3[3]; 4[5, 15]; 5[6, 15, 16]; 6[30]; 7[15, 16, 18, 31]; | 1[32]; 2[33]; | 1[34]; 2[35]; 3[36]; 4[37]; 5[38]; | 1[39]; 2[40]; 3[41]; 4[42]; | 1[43]; | 1[44]; | 1[45]; | 1[46]; 2[4]; | 1[47]; 2[48]; | 1[49];2[50];3[48]; | 1[51]; | 1[52]; | 1[52]; | 1[53]; |
| --- | --- | --- | --- | --- | --- | --- | --- | --- | --- | --- | --- | --- | --- | --- | --- | --- | --- | --- | --- | --- |
|  | **Stage** |  | 2,7min |  | 2,10min |  | 1,7,0-8min;2-5,0-10min;6, 0-15min |  |  | 1,20min;4,30min |  |  | 1,15min | 1-2,0-30min | 1-2,5 days | 1-3, 5 days | 1,5 days | 1,5 days | 1,5 days | 1,1.5-3 days |
|  | **Species** | 1,dog | 1,human; 2,guinea pig | 1,guinea pig | 1,human; 2-4,guinea pig; 5,rabbit | 1-2,rabbit; 3-4,human | 1,pig; 2-3,5,7,rabbit;  4,6,guinea pig | 1,chick; 2,rabbit | 1,pig; 2-4,guinea-pig; 5,canine | 1-3,guinea pig;4,rat | 1,pig | 1,rat | 1,rabbit | 1,swine; 2, rabbit | 1,2, dog; | 1-3, dog; | 1,dog | 1,canine | 1,canine |  |
|  | **Experimental data** | 1,I_Na_↓22.7%, mV_1/2_-1.7mV | 1,I_NaL_↑; 2,I_NaL_↑50% | 1,I_Ks_↓21.9 ± 1.8% | 1,I_CaL_↓10%~20%;2-4,I_CaL_↓20%;5,I_CaL_↓ | 1,I_to_↓50%;2-3, I_to_↓;4, rV_1/2_-7.2 mV, sV_1/2_-13.7mV | 1,3.3-11mM;2,5-7mM;3,10 mM;4,4-9 mM;5,4-10mM;6,5-14mM;7,5-10mM | 1,I_NaK_↓54%; 2,I_NaK_↓ | 1,4,I_NaCa_↓; 2,I_NaCa_↓54%; 3,I_NaCa_↓30%; | 1, I_CaL_↓51%; 2-4, I_CaL_↓ | 1,I_bCa_↑ | 1,I_rel_↓35% | 1,I_up_↓29% | 1, 4-10mM; 2, [K^+^]_o_↑ | 1,I_Na_↓62%; 2,I_Na_↓ | 1,I_CaL_↓36%; 2,I_CaL_↓38%; 3,I_CaL_↓ | 1,I_to_↓63% | 1,I_Kr_↓70% | 1,I_Ks_↓80% | 1,[K^+^]_o_↑ |
|  | **Simulation** | ×0.887, mV_1/2_-1.7 | ×1.5 | ×0.781 | ×0.8 | ×0.5, rV_1/2_-7.2, sV_1/2_-13.7mV | 8mM | ×0.46 | ×0.6 | ×0.5 | ×1.3 | ×0.65 | ×0.71 | 8mM | ×0.38 | ×0.62 | ×0.37 | ×0.3 | ×0.2 | 8mM |
|  | **Parameters** | G_Na_ | G_NaL_ | G_Ks_ | G_CaL_ | G_to_ | [K^+^]_o_ | G_NaK_ | G_NaCa_ | G_CaL_ | G_bCa_ | G_rel_ | G_up_ | [K^+^]_o_ | G_Na_ | G_CaL_ | G_to_ | G_Kr_ | G_Ks_ | [K^+^]_o_ |
|  | **Phases** | **Ischemia 1a** | | | | | | **Ischemia 1b** | | | | | | | **MI** | | | | | |

Notes: mV_1/2_ is half activation potential of I_Na_; rV_1/2_ is half activation potential of I_to_, sV_1/2_ is half inactivation potential of I_to_. The change of current amplitude was mainly mimicked by modifying the conductivity value, and the left and right shifts of activation or inactivation curve are simulated by modifying the corresponding half activation or inactivation potential (V_1/2_) of the activation or inactivation curves. It is worth noting that, for I_Na_, the movement of activation curve also affects the current amplitude. Therefore, the conductance of I_Na_ and activation half action potential were adjusted simultaneously to reproduce the change of I_Na_ current amplitude consistent with the experimental data. The prefixes 1, 2, and so on correspond to the corresponding reference numbers, respectively.

**B Stimulation protocols**

Stimulation protocols in Fig 2 and Figs A-B, H-J in S1 Text.

The standard S1-S2 protocol was used in Fig 2 and Figs A-B, H-J in S1 Text with a stimulation strength of −86.2 pA/pF and a stimulus duration of 1 ms. In the S1-S2 protocol, 100 S1 stimuli with the interval of 1000 ms were applied for reaching a steady state.

Stimulation protocols in Fig 3 and Fig D in S1 Text.

The standard S1-S2 protocol was used in Fig 3 and Fig D in S1 Text with a stimulation strength of −120 pA/pF and a stimulus duration of 3 ms. In the S1-S2 protocol, five S1 stimuli with the interval of 1000 ms were applied on the leftmost three columns of nodes before S2 stimulus, which ensures the tissue reaching a steady state. S2 stimulus was applied in the lower left corner with the size of 300×300 cells in Fig 3B and Fig D in S1 Text. S2 stimulus was applied in the (Dark Red) lower left corner or (Orange) upper left corner in Fig 3C.

Stimulation protocols in Fig 4 and Fig E in S1 Text.

The standard S1-S2 protocol was used in Fig 4 and Fig E in S1 Text with a stimulation strength of −120 pA/pF and a stimulus duration of 3 ms. In the S1-S2 protocol, five S1 stimuli with the interval of 1000 ms were applied on the leftmost three columns of nodes before S2 stimulus, which ensures the tissue reaching a steady state. S2 stimulus was applied in the lower left and upper left corner with the size of 300×300 cells in Fig 4 and Fig E in S1 Text.

Stimulation protocols in Fig 5.

The standard S1-S2 protocol was used in Fig 5A with a stimulation strength of −120 pA/pF and a stimulus duration of 3 ms. In the S1-S2 protocol, five S1 stimuli with the interval of 1000 ms were applied on the leftmost three columns of nodes before S2 stimulus, which ensures the tissue reaching a steady state. S2 stimulus was applied in the leftmost three columns with the size of 3×600 cells.

The dynamic protocol was used in Fig 5B with a stimulation strength of −120 pA/pF and a stimulus duration of 3 ms. In the dynamic protocol, stimuli were applied on the leftmost three columns with the size of 3×600 cells with a pacing cycle of 250ms.

Stimulation protocols in Fig 6 and Fig L in S1 Text.

The dynamic protocol was used in Fig 6 and Fig L in S1 Text with a stimulation strength of −120 pA/pF and a stimulus duration of 3 ms. In the dynamic protocol, stimuli were applied on the leftmost three columns with the size of 3×600 cells with pacing cycles of 250ms in Fig 6 and 290ms in Fig L in S1 Text.

Stimulation protocols in Fig 7, Fig 8, and Fig F in S1 Text.

The dynamic protocol was used in Fig 7, Fig 8, and Fig F in S1 Text with a stimulation strength of −120 pA/pF and a stimulus duration of 3 ms. In the dynamic protocol, stimuli were applied on a circular sector at the upper left corner with a radius of 5 nodes with a pacing cycle of 420ms.

Stimulation protocols in Fig 9 and Fig G in S1 Text.

In the 3D ventricular tissue as shown in Fig 9 and Fig G in S1 Text, three stimuli were applied on a small cubic tissue with the size of 10×10×10 nodes in intramyocardial region with a stimulation strength of −120 pA/pF and a stimulus duration of 2 ms.

Stimulation protocols in Fig 10, Fig K and Fig M in S1 Text.

The standard S1-S2 protocol was used in Fig 10, Fig K and Fig M in S1 Text with a stimulation strength of −120 pA/pF and a stimulus duration of 3 ms. In the S1-S2 protocol, five S1 stimuli with the interval of 1000 ms were applied on the leftmost three columns of nodes before S2 stimulus, which ensures the tissue reaching a steady state. S2 stimulus was applied in the leftmost three columns with the size of 3×600 cells.

Stimulation protocols in Fig 2C and Fig C in S1 Text.

The dynamic protocol was used in Fig 2C and Fig C in S1 Text with a stimulation strength of −120 pA/pF and a stimulus duration of 3 ms. In the dynamic protocol, stimuli were applied on the leftmost three cells.

**C Supplementary simulation results**

In addition, the 2D tissue shown in Fig 1Ai (right panel) can be surrounded by a region of normal tissue as shown in S13A Fig. The upper area or rightmost area of the tissue are chosen as normal area due to closing to the blood vessel. Meanwhile, the changes of ion currents and ion concentrations in these three pathological stages (ischemia 1a, 1b and MI) were set with gradient rather than with abrupt changes at the borders. Then, we carried out corresponding simulations on these two different tissues. The results showed that, when the width of the normal region L1 is less than 200*0.25 mm as shown in S13Ai Fig, the VWs have barely changed, comparing to the VWs shown in Fig 10B. The main reason for this is that the reentrant rotors were mainly located in the ischemia 1b and MI regions below the tissue. When the right region of the tissue was set to normal state, as shown in S13Aii Fig, there was also almost no change in the size of the VW when the width of the normal region L2 is less than 80*0.25 mm. If the width of the normal region further increases, the lower bound of the VW corresponding to reentry is gradually raised, resulting in a decrease in the overall width of VWs for conditions with region-wise heterogeneity and gradient-wise heterogeneity of I_Na_ shown in S13C Fig (the width of the normal region L2 is equal to 140*0.25mm). This may be attributed to a simple fact that the pathological regions become smaller after introducing a normal region.


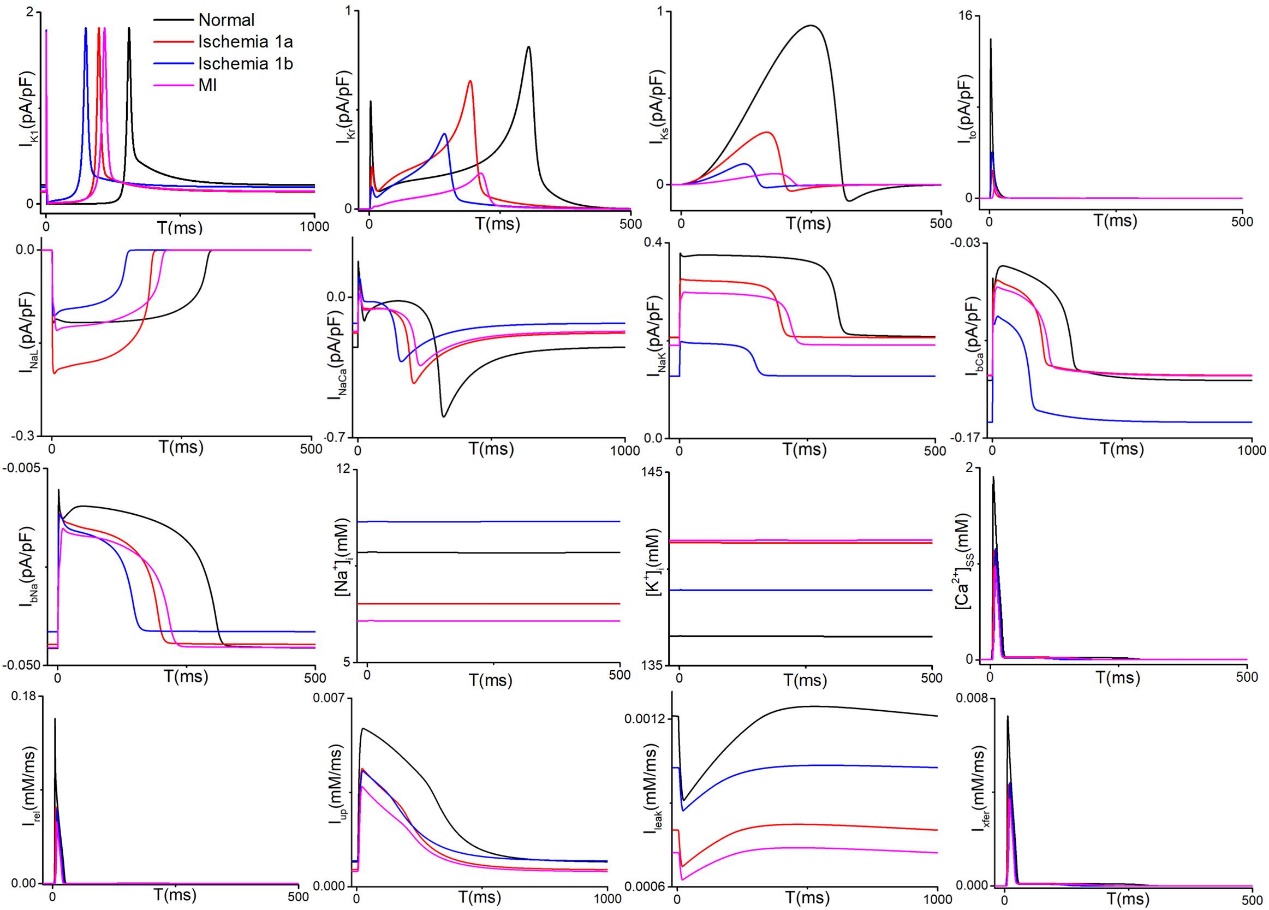


**Fig B.** Variations of electrophysiological characteristics of other ion currents and concentration of single cells in normal, ischemia1a, ischemia1b, and MI conditions.


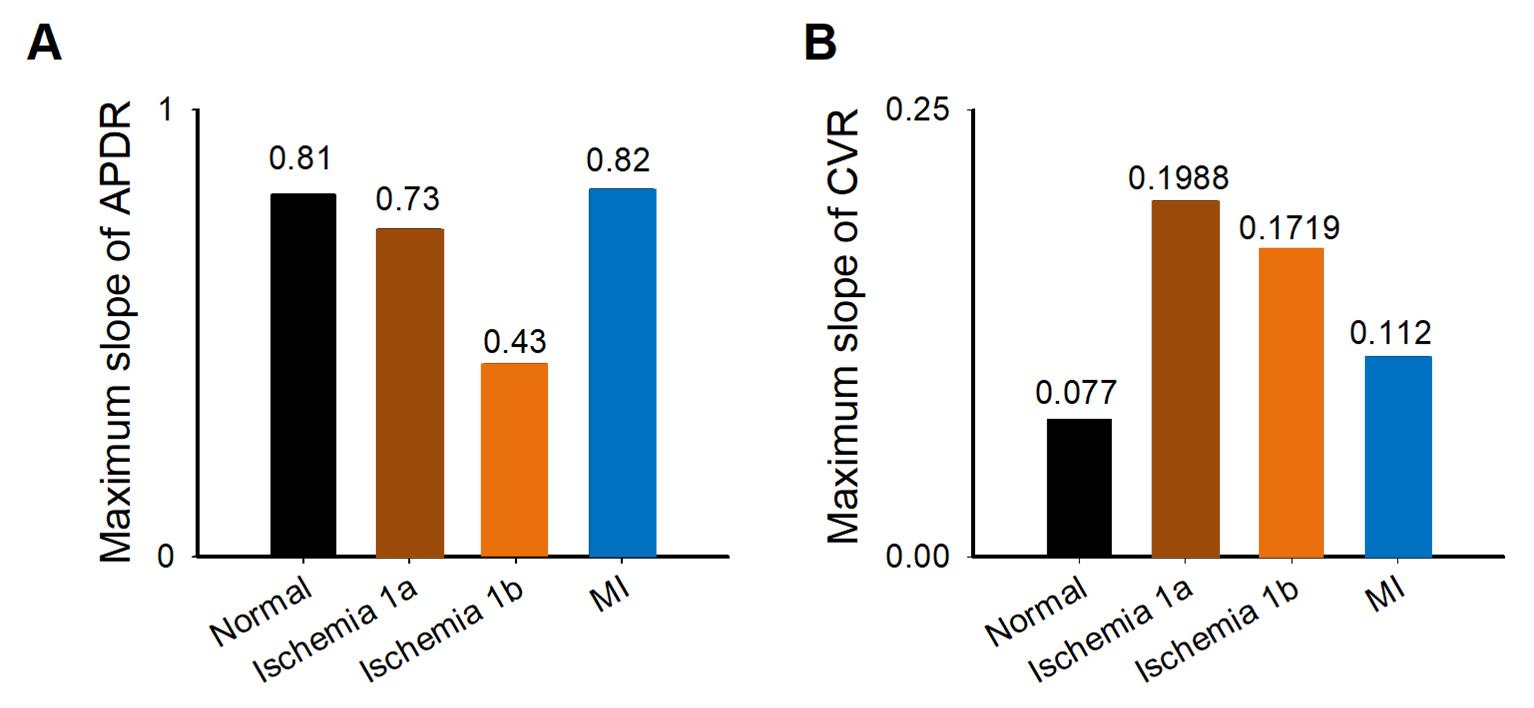


**Fig C.** Maximum slopes of APD and CV restitution curves in four conditions (normal, ischemia 1a, 1b and MI). (A) Maximum slopes of APD restitution curves; (B) Maximum slopes of CV restitution curves.


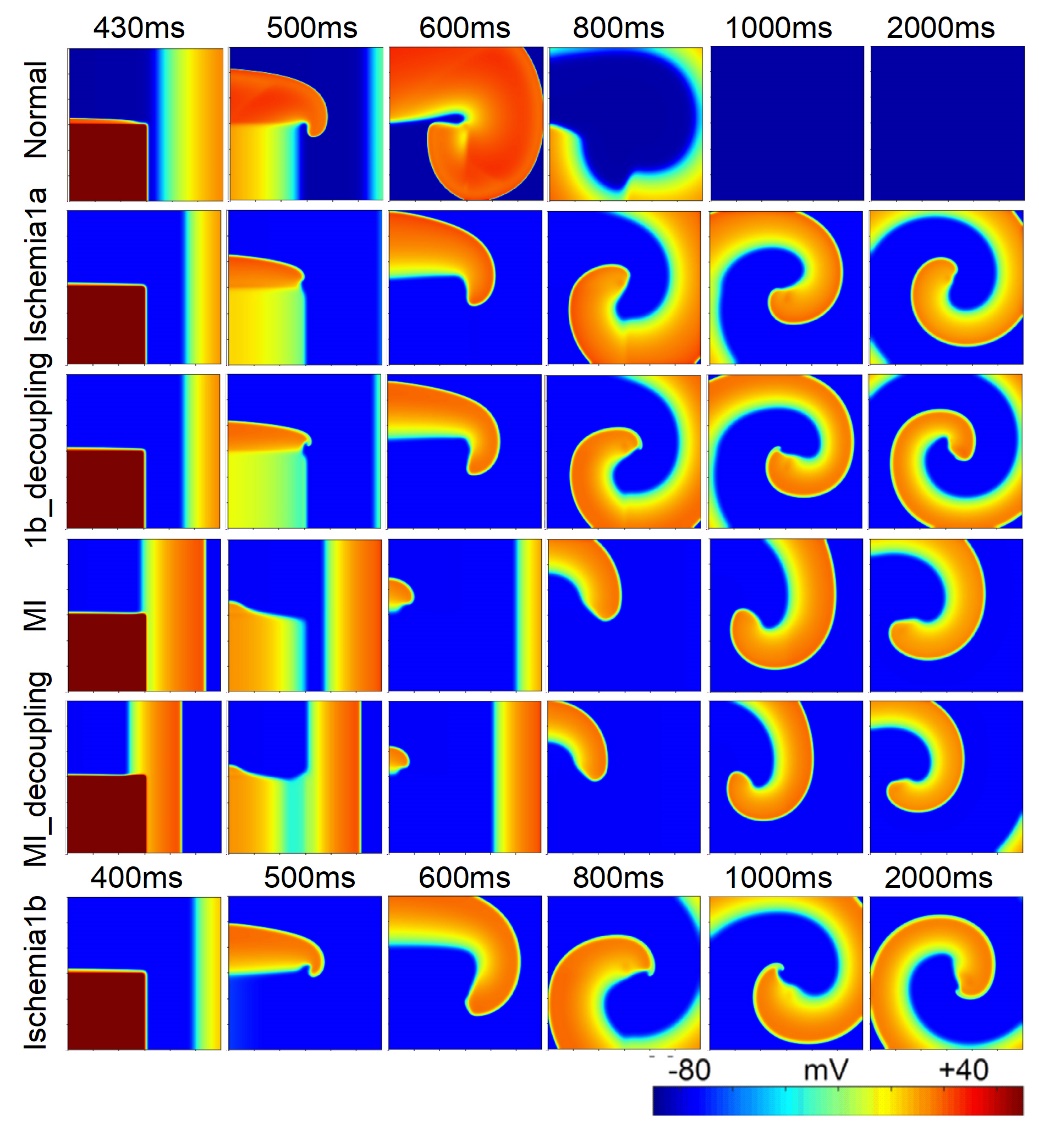


**Fig D.** Wave propagation in 2D homogenous tissues: normal, ischemia 1a, ischemia 1b, MI, decoupled 1b and MI. (The time interval of S2 stimulation was 390ms in ischemia 1b condition and the rest was 420ms.)


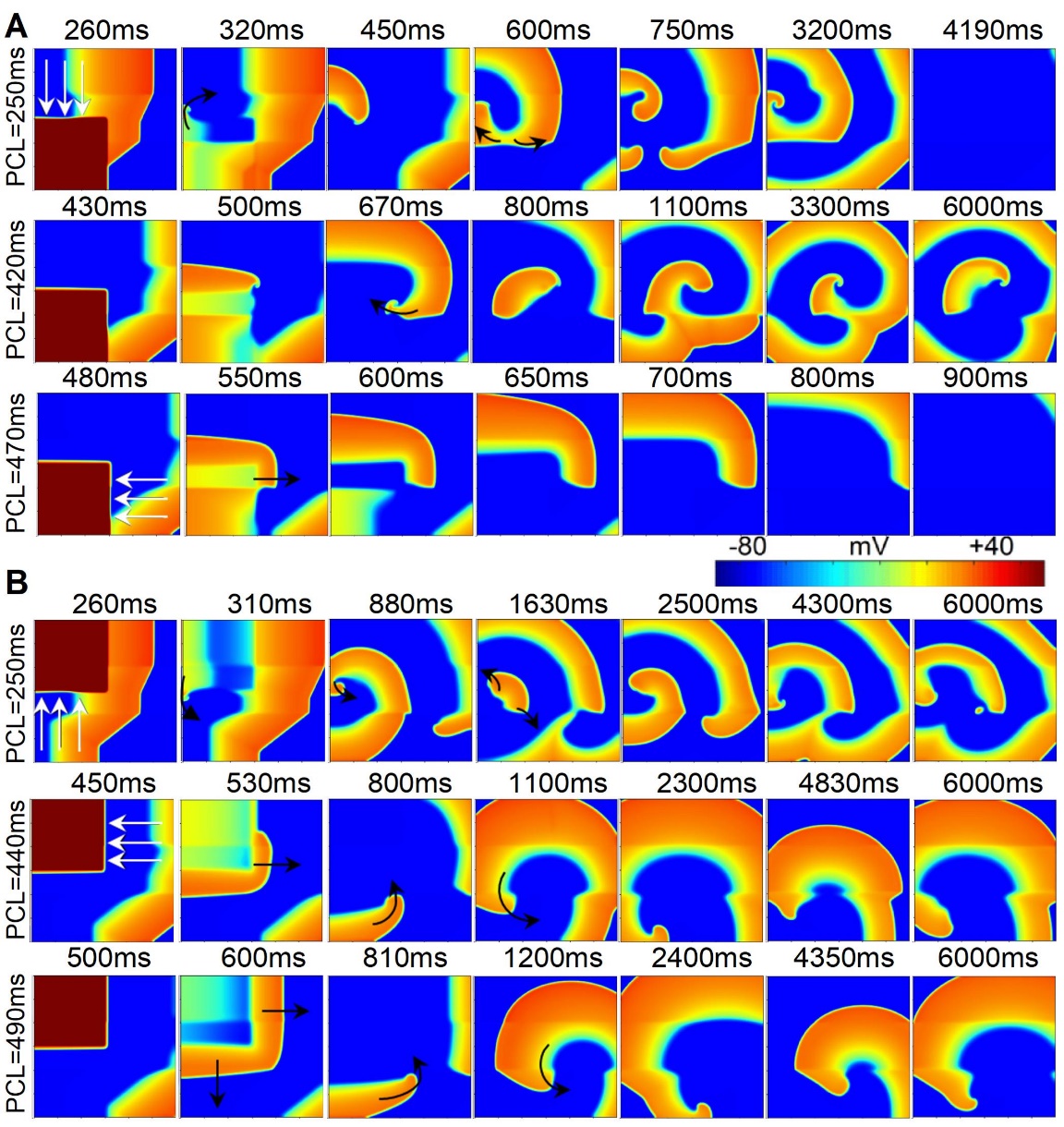


**Fig E.** Wave propagation in the 2D tissue where ischemia 1a, decoupled ischemia 1b, and decoupled MI distributed horizontally (Fig 1Ai, right panel) using the S1-S2 protocol when the S2 stimulus was applied in the (A) lower left or (B) upper left corner.


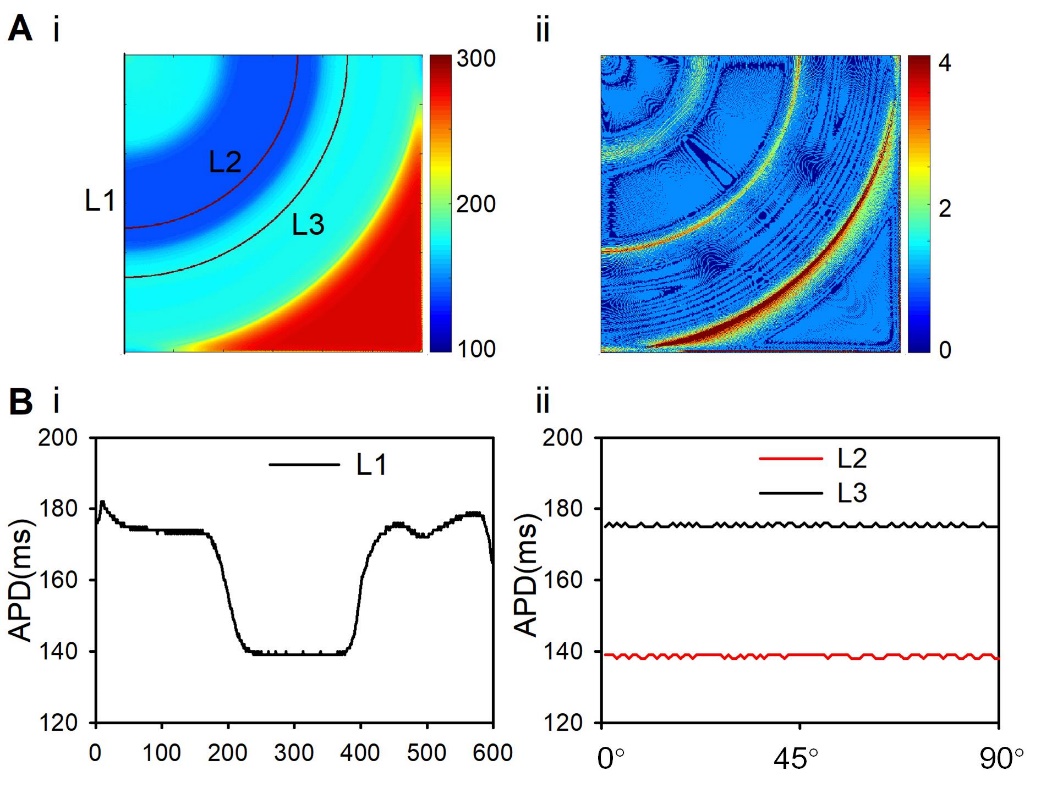


**Fig F.** APD distribution and APD of all cells along lines L1, L2 and L3 of the fifth stimulation in the 2D tissue where ischemia 1a, decoupled 1b, and decoupled MI distributed circularly (Fig 1Aii), when the upper left corner stimulation was applied using dynamic stimulation protocol with a pacing cycle of 420ms. (A) (i) APD distribution in the fifth stimulation in the 2D tissue. (ii) The maximum APD difference between each cell and its neighbors in the 2D tissue. (B) (i) APD of all cells along the border (line L1) in the 2D tissue. (ii) APD of all cells along the line L2 (with a radius of 350*0.25mm) and L3 (with a radius of 450*0.25mm) in the 2D tissue.


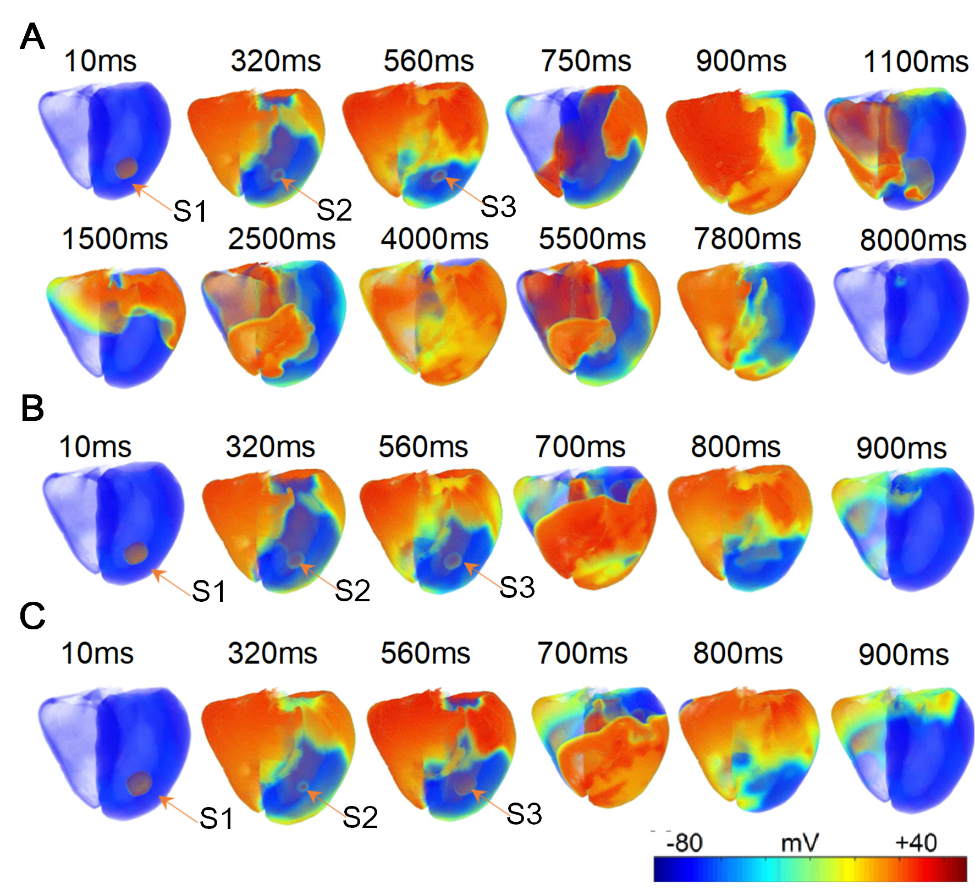


**Fig G.** Wave propagation in the 3D ventricular tissue (A) with ischemia 1a, (B) with ischemia 1b, or (C) with MI areas (with stimulus intervals 310ms and 240ms, respectively).


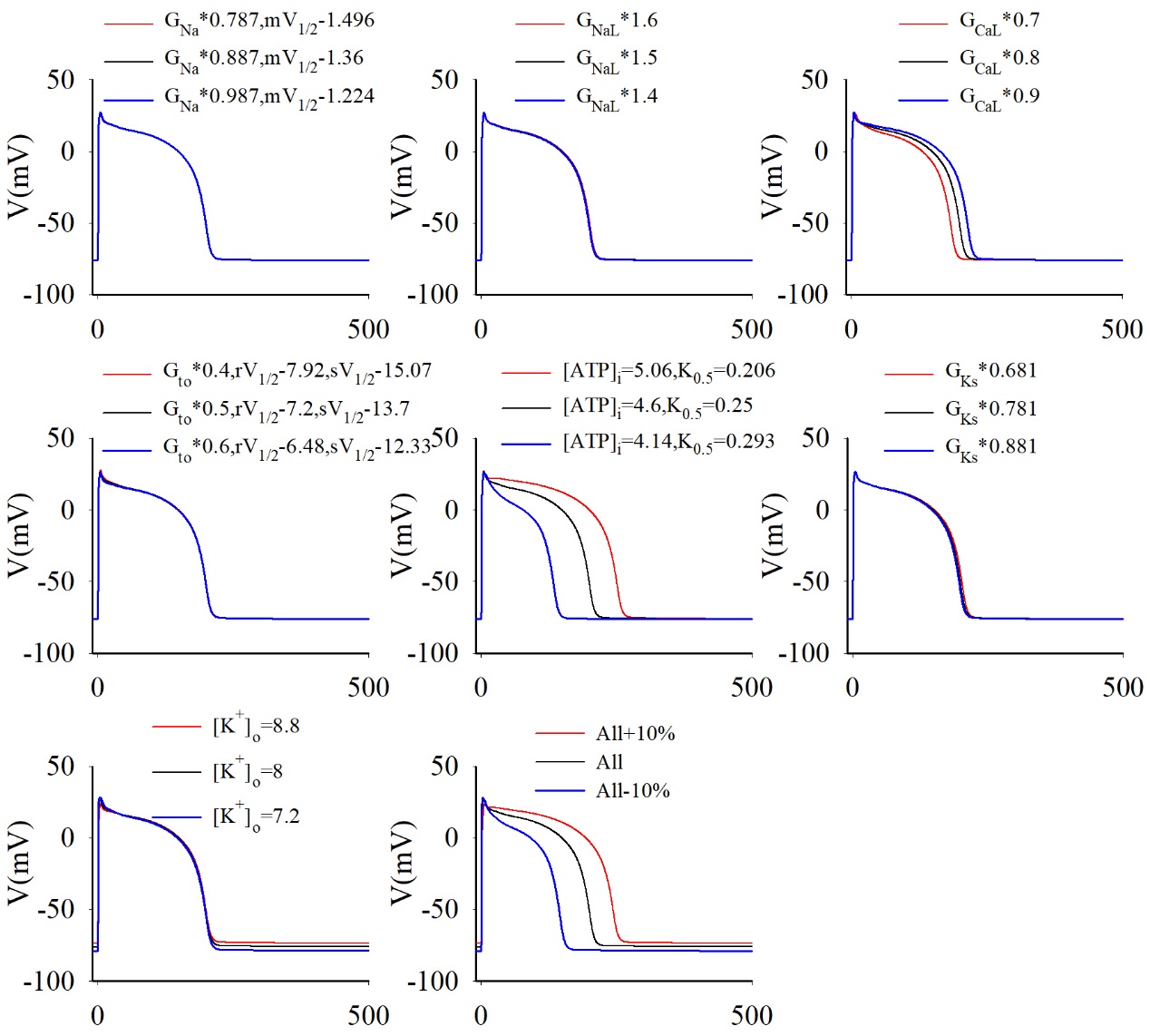


**Fig H.** The change of cellular AP when each parameter changes alone and simultaneously in the single-cell model of ischemia 1a.


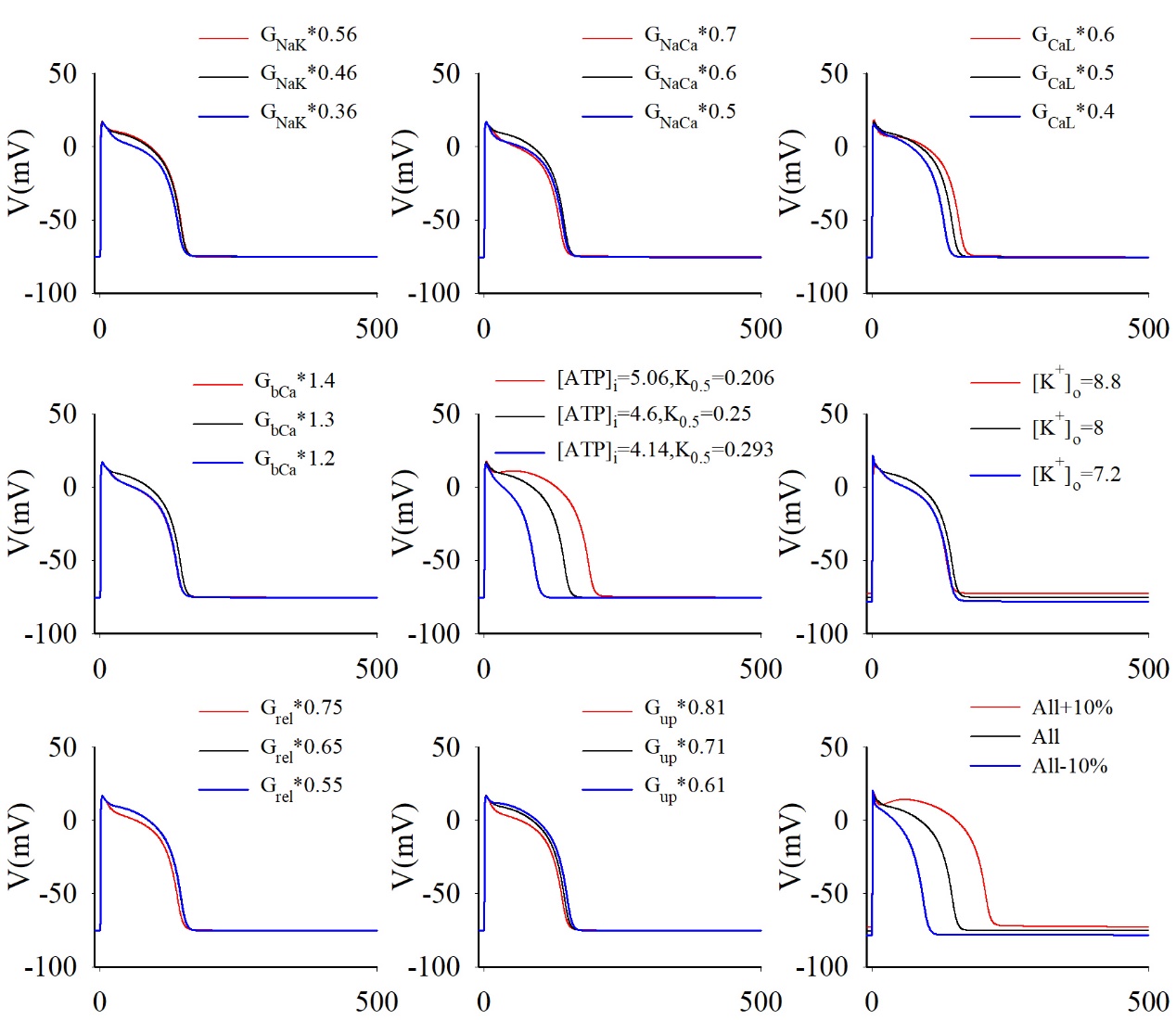


**Fig I.** The change of cellular AP when each parameter changes alone and simultaneously in the single-cell model of ischemia 1b.


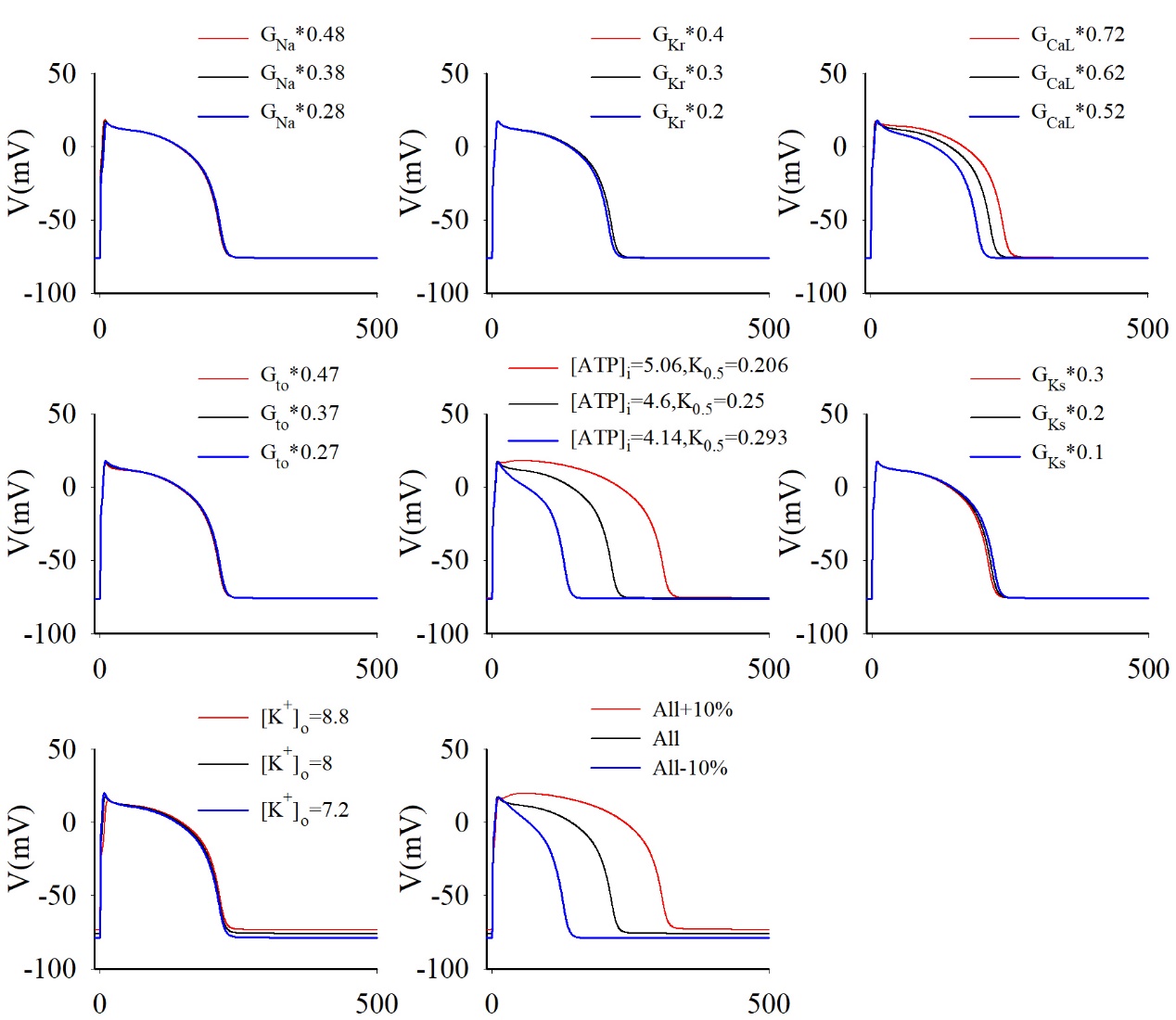


**Fig J.** The change of cellular AP when each parameter changes alone and simultaneously in the single-cell model of MI.


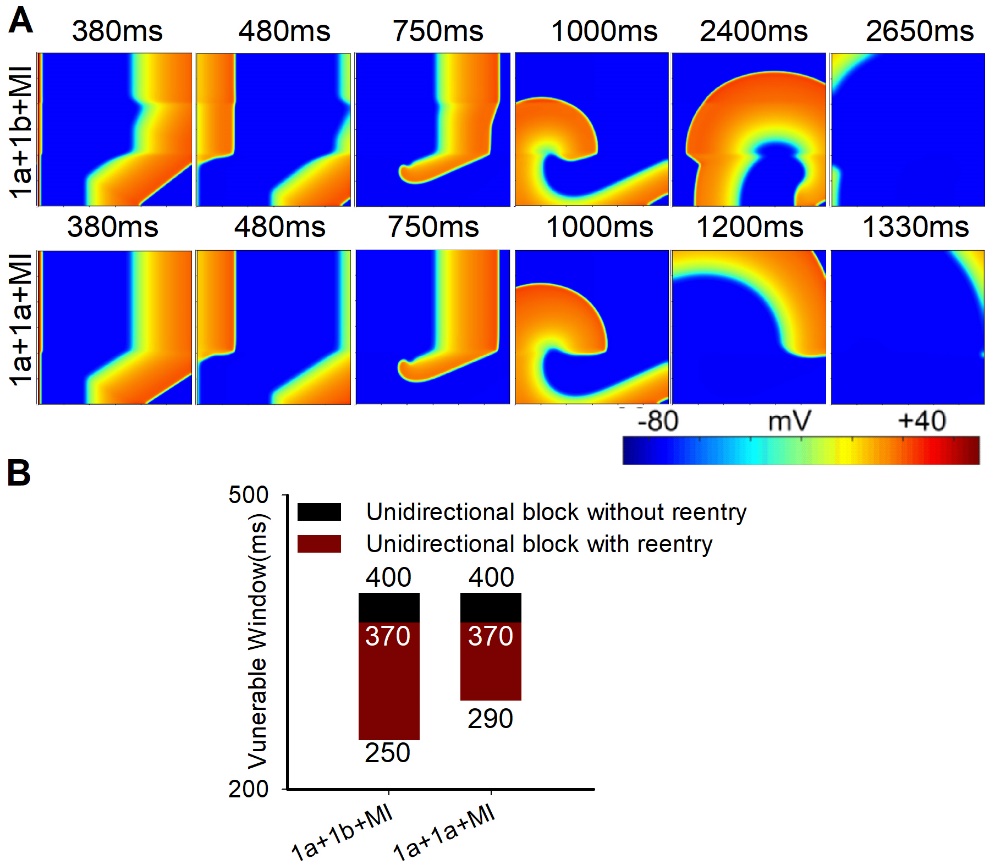


**Fig K.** Wave propagation in the 2D tissue where ischemia 1a, decoupled 1b, and decoupled MI distributed horizontally (Fig 1Ai, right panel) before and after ischemia 1b area was replaced with ischemia 1a area, when the leftmost stimulation was applied using the S1-S2 protocol.

(A) Wave propagation in the 2D tissue. (B) VWs in the 2D tissue.


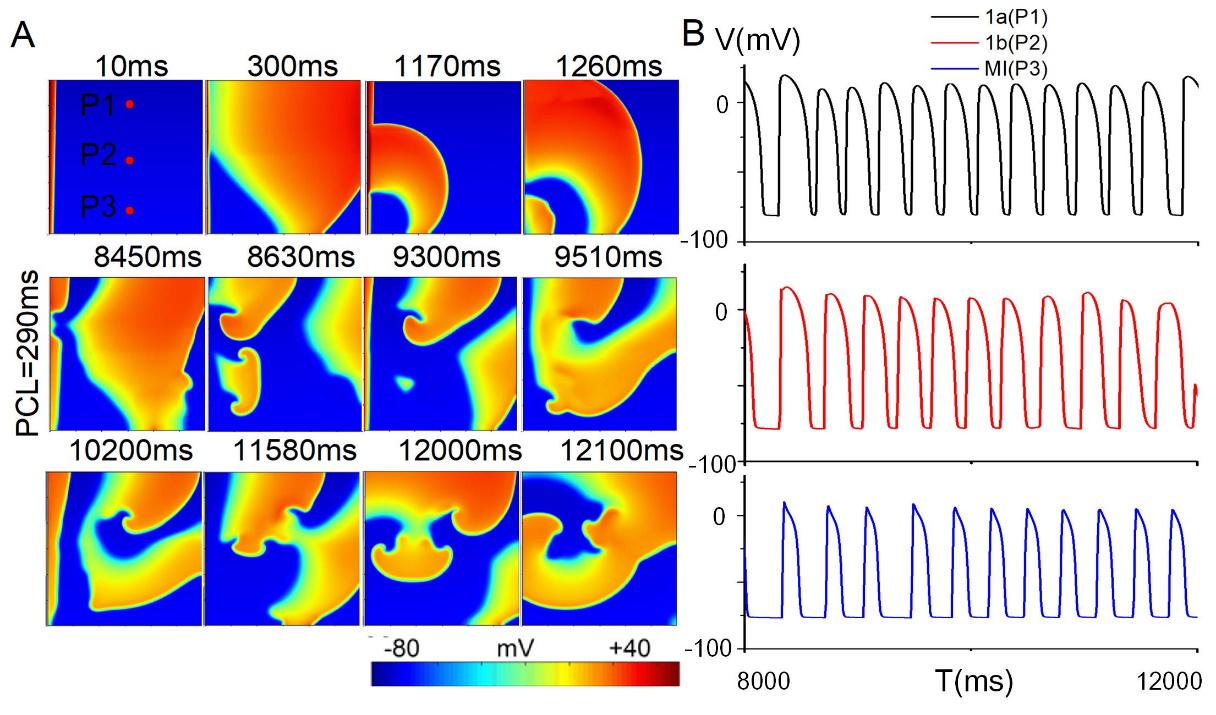


**Fig L.** Wave propagation in the 2D tissue where ischemia 1a, 1b, and MI distributed horizontally (Fig 1Ai, right panel) with gradient distribution of all parameters and action potentials of points P1, P2, and P3 when the leftmost stimulation was applied with a pacing cycle of 290ms using the dynamic stimulation protocol. (A) Wave propagation in the 2D tissue. (B) action potentials of points P1, P2, and P3 when the leftmost stimulation was applied with a pacing cycle of 290ms.


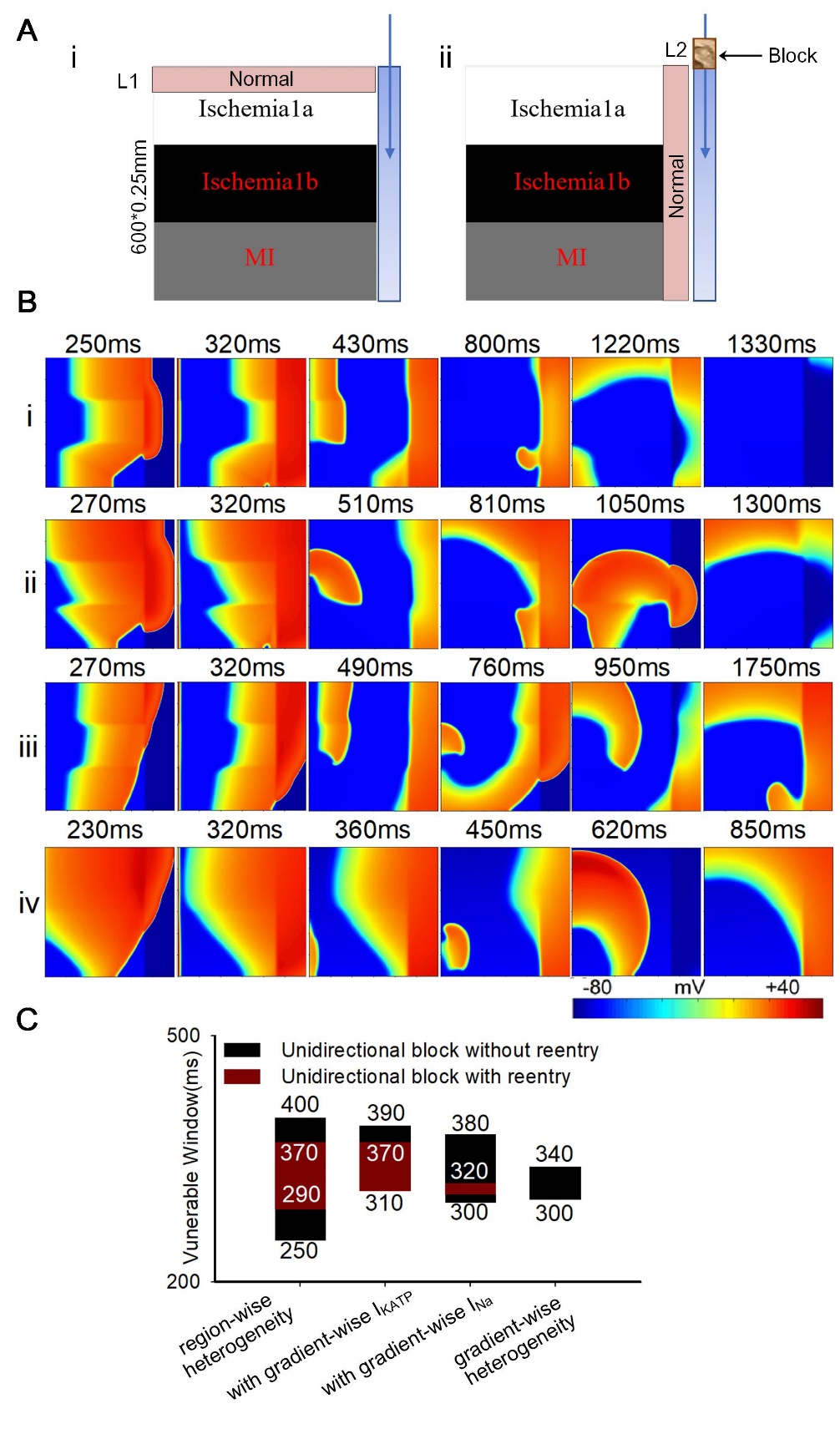


**Fig M.** Tissue architecture of two new 2D ideal tissues, wave propagation and the distribution of VWs in the 2D tissue with ischemia 1a, 1b, and MI when the leftmost stimulation was applied in four different conditions using S1-S2 protocol. (A) Tissue architecture of two new distribution (Ai and Aii) of 2D ideal tissues with different pathological areas and normal areas. (B) Wave propagation in the 2D tissue (L2 is equal to 140*0.25mm) in four different conditions with the S2 stimulation interval of 310ms. (C) The distribution of VWs in the 2D tissue (L2 is equal to 140*0.25mm) in four different conditions: (i) region-wise heterogeneity, (ii) with gradient-wise I_KATP_, (iii) with gradient-wise I_Na_, (iv) gradient-wise heterogeneity of all parameters.

**References**

1. Watanabe I, Kanda A, Engle CL, Gettes LS. Comparison of the effects of regional ischemia and hyperkalemia on the membrane action potentials of the in situ pig heart. Experimental Cardiology Group, University of North Carolina at Chapel Hill. J Cardiovasc Electrophysiol. 1997;8(11):1229-36. doi: 10.1111/j.1540-8167.1997.tb01012.x. PMID: 9395164.

2. Vermeulen JT, Tan HL, Rademaker H, Schumacher CA, Loh P, Opthof T, et al. Electrophysiologic and extracellular ionic changes during acute ischemia in failing and normal rabbit myocardium. J Mol Cell Cardiol. 1996;28(1):123-31. doi: 10.1006/jmcc.1996.0012. PMID: 8745220.

3. Weiss J, Shine KI. [K+]o accumulation and electrophysiological alterations during early myocardial ischemia. Am J Physiol. 1982;243(2):H318-27. doi: 10.1152/ajpheart.1982.243.2.H318. PMID: 7114241.

4. Wilde AA, Escande D, Schumacher CA, Thuringer D, Mestre M, Fiolet JW, et al. Potassium accumulation in the globally ischemic mammalian heart. A role for the ATP-sensitive potassium channel. Circ Res. 1990;67(4):835-43. doi: 10.1161/01.res.67.4.835. PMID: 2119912.

5. Weiss JN, Venkatesh N, Lamp ST. ATP-sensitive K+ channels and cellular K+ loss in hypoxic and ischaemic mammalian ventricle. J Physiol. 1992;447:649-73. doi: 10.1113/jphysiol.1992.sp019022. PMID: 1593462.

6. Weiss J, Shine KI. Effects of heart rate on extracellular [K+] accumulation during myocardial ischemia. Am J Physiol. 1986;250(6 Pt 2):H982-91. doi: 10.1152/ajpheart.1986.250.6.H982. PMID: 3717369.

7. Ferrero JM, Jr., Saiz J, Ferrero JM, Thakor NV. Simulation of action potentials from metabolically impaired cardiac myocytes. Role of ATP-sensitive K+ current. Circ Res. 1996;79(2):208-21. doi: 10.1161/01.res.79.2.208. PMID: 8755997.

8. Shaw RM, Rudy Y. Electrophysiologic effects of acute myocardial ischemia: a theoretical study of altered cell excitability and action potential duration. Cardiovasc Res. 1997;35(2):256-72. doi: 10.1016/s0008-6363(97)00093-x. PMID: 9349389.

9. Murphy L, Renodin D, Antzelevitch C, Di Diego JM, Cordeiro JM. Extracellular proton depression of peak and late Na(+) current in the canine left ventricle. Am J Physiol Heart Circ Physiol. 2011;301(3):H936-44. doi: 10.1152/ajpheart.00204.2011. PMID: 21685271.

10. Sutton PM, Taggart P, Opthof T, Coronel R, Trimlett R, Pugsley W, et al. Repolarisation and refractoriness during early ischaemia in humans. Heart. 2000;84(4):365-9. doi: 10.1136/heart.84.4.365. PMID: 10995401.

11. Dutta S, Minchole A, Quinn TA, Rodriguez B. Electrophysiological properties of computational human ventricular cell action potential models under acute ischemic conditions. Prog Biophys Mol Biol. 2017;129:40-52. doi: 10.1016/j.pbiomolbio.2017.02.007. PMID: 28223156.

12. Pollard AE, Cascio WE, Fast VG, Knisley SB. Modulation of triggered activity by uncoupling in the ischemic border. A model study with phase 1b-like conditions. Cardiovasc Res. 2002;56(3):381-92. doi: 10.1016/s0008-6363(02)00598-9. PMID: 12445879.

13. Gardner PI, Ursell PC, Fenoglio JJ, Jr., Wit AL. Electrophysiologic and anatomic basis for fractionated electrograms recorded from healed myocardial infarcts. Circulation. 1985;72(3):596-611. doi: 10.1161/01.cir.72.3.596. PMID: 4017211.

14. Ursell PC, Gardner PI, Albala A, Fenoglio JJ, Jr., Wit AL. Structural and electrophysiological changes in the epicardial border zone of canine myocardial infarcts during infarct healing. Circ Res. 1985;56(3):436-51. doi: 10.1161/01.res.56.3.436. PMID: 3971515.

15. Jones DK, Peters CH, Tolhurst SA, Claydon TW, Ruben PC. Extracellular proton modulation of the cardiac voltage-gated sodium channel, Nav1.5. Biophys J. 2011;101(9):2147-56. doi: 10.1016/j.bpj.2011.08.056. PMID: 22067152.

16. Zheng J, Ma J, Zhang P, Hu L, Fan X, Tang Q. Milrinone inhibits hypoxia or hydrogen dioxide-induced persistent sodium current in ventricular myocytes. Eur J Pharmacol. 2009;616(1-3):206-12. doi: 10.1016/j.ejphar.2009.06.021. PMID: 19549513.

17. Hool LC. Differential regulation of the slow and rapid components of guinea-pig cardiac delayed rectifier K+ channels by hypoxia. J Physiol. 2004;554(Pt 3):743-54. doi: 10.1113/jphysiol.2003.055442. PMID: 14634203.

18. Fernandez-Morales JC, Hua W, Yao Y, Morad M. Regulation of Ca(2+) signaling by acute hypoxia and acidosis in cardiomyocytes derived from human induced pluripotent stem cells. Cell Calcium. 2019;78:1-14. doi: 10.1016/j.ceca.2018.12.006. PMID: 30579812.

19. Sato R, Noma A, Kurachi Y, Irisawa H. Effects of intracellular acidification on membrane currents in ventricular cells of the guinea pig. Circ Res. 1985;57(4):553-61. doi: 10.1161/01.res.57.4.553. PMID: 2412722.

20. Hool LC. Hypoxia increases the sensitivity of the L-type Ca(2+) current to beta-adrenergic receptor stimulation via a C2 region-containing protein kinase C isoform. Circ Res. 2000;87(12):1164-71. doi: 10.1161/01.res.87.12.1164. PMID: 11110774.

21. Irisawa H, Sato R. Intra- and extracellular actions of proton on the calcium current of isolated guinea pig ventricular cells. Circ Res. 1986;59(3):348-55. doi: 10.1161/01.res.59.3.348. PMID: 2429781.

22. Saegusa N, Moorhouse E, Vaughan-Jones RD, Spitzer KW. Influence of pH on Ca(2)(+) current and its control of electrical and Ca(2)(+) signaling in ventricular myocytes. J Gen Physiol. 2011;138(5):537-59. doi: 10.1085/jgp.201110658. PMID: 22042988.

23. Ferrero JM, Trenor B, Romero L. Multiscale computational analysis of the bioelectric consequences of myocardial ischaemia and infarction. Europace. 2014;16(3):405-15. doi: 10.1093/europace/eut405. PMID: 24569895.

24. Saegusa N, Garg V, Spitzer KW. Modulation of ventricular transient outward K(+) current by acidosis and its effects on excitation-contraction coupling. Am J Physiol Heart Circ Physiol. 2013;304(12):H1680-96. doi: 10.1152/ajpheart.00070.2013. PMID: 23585132.

25. Ogbaghebriel A, Shrier A. Inhibition of metabolism abolishes transient outward current in rabbit atrial myocytes. Am J Physiol. 1994;266(1 Pt 2):H182-90. doi: 10.1152/ajpheart.1994.266.1.H182. PMID: 8304498.

26. Verkerk AO, Veldkamp MW, van Ginneken AC, Bouman LN. Biphasic response of action potential duration to metabolic inhibition in rabbit and human ventricular myocytes: role of transient outward current and ATP-regulated potassium current. J Mol Cell Cardiol. 1996;28(12):2443-56. doi: 10.1006/jmcc.1996.0237. PMID: 9004161.

27. Stengl M, Carmeliet E, Mubagwa K, Flameng W. Modulation of transient outward current by extracellular protons and Cd2+ in rat and human ventricular myocytes. J Physiol. 1998;511 ( Pt 3):827-36. doi: 10.1111/j.1469-7793.1998.827bg.x. PMID: 9714863.

28. Arevalo H, Plank G, Helm P, Halperin H, Trayanova N. Tachycardia in post-infarction hearts: insights from 3D image-based ventricular models. PLoS One. 2013;8(7):e68872. doi: 10.1371/journal.pone.0068872. PMID: 23844245.

29. Deng D, Arevalo HJ, Prakosa A, Callans DJ, Trayanova NA. A feasibility study of arrhythmia risk prediction in patients with myocardial infarction and preserved ejection fraction. Europace. 2016;18(suppl 4):iv60-iv6. doi: 10.1093/europace/euw351. PMID: 28011832.

30. Kleber AG. Resting membrane potential, extracellular potassium activity, and intracellular sodium activity during acute global ischemia in isolated perfused guinea pig hearts. Circ Res. 1983;52(4):442-50. doi: 10.1161/01.res.52.4.442. PMID: 6831660.

31. Owens LM, Fralix TA, Murphy E, Cascio WE, Gettes LS. Correlation of ischemia-induced extracellular and intracellular ion changes to cell-to-cell electrical uncoupling in isolated blood-perfused rabbit hearts. Experimental Working Group. Circulation. 1996;94(1):10-3. doi: 10.1161/01.cir.94.1.10. PMID: 8964108.

32. Ikenouchi H, Zhao L, McMillan M, Hammond EM, Barry WH. ATP depletion causes a reversible decrease in Na+ pump density in cultured ventricular myocytes. Am J Physiol. 1993;264(4 Pt 2):H1208-14. doi: 10.1152/ajpheart.1993.264.4.H1208. PMID: 8386481.

33. Bersohn MM. Sodium pump inhibition in sarcolemma from ischemic hearts. J Mol Cell Cardiol. 1995;27(8):1483-9. doi: 10.1016/s0022-2828(95)90161-2. PMID: 8523412.

34. Doering AE, Lederer WJ. The mechanism by which cytoplasmic protons inhibit the sodium-calcium exchanger in guinea-pig heart cells. J Physiol. 1993;466:481-99. PMID: 8410703.

35. Shigematsu S, Arita M. Anoxia depresses sodium-calcium exchange currents in guinea-pig ventricular myocytes. J Mol Cell Cardiol. 1999;31(4):895-906. doi: 10.1006/jmcc.1998.0929. PMID: 10329216.

36. Egger M, Niggli E. Paradoxical block of the Na+-Ca2+ exchanger by extracellular protons in guinea-pig ventricular myocytes. J Physiol. 2000;523 Pt 2:353-66. doi: 10.1111/j.1469-7793.2000.t01-1-00353.x. PMID: 10699080.

37. Earm YE, Irisawa H. Effects of pH on the Na-Ca exchange current in single ventricular cells of the guinea pig. Jpn Heart J. 1986;27 Suppl 1:153-8. PMID: 2434676.

38. Philipson KD, Bersohn MM, Nishimoto AY. Effects of pH on Na+-Ca2+ exchange in canine cardiac sarcolemmal vesicles. Circ Res. 1982;50(2):287-93. doi: 10.1161/01.res.50.2.287. PMID: 7055859.

39. Cordeiro JM, Howlett SE, Ferrier GR. Simulated ischaemia and reperfusion in isolated guinea pig ventricular myocytes. Cardiovasc Res. 1994;28(12):1794-802. doi: 10.1093/cvr/28.12.1794. PMID: 7867032.

40. Prod'hom B, Pietrobon D, Hess P. Interactions of protons with single open L-type calcium channels. Location of protonation site and dependence of proton-induced current fluctuations on concentration and species of permeant ion. J Gen Physiol. 1989;94(1):23-42. doi: 10.1085/jgp.94.1.23. PMID: 2553858.

41. Kaibara M, Kameyama M. Inhibition of the calcium channel by intracellular protons in single ventricular myocytes of the guinea-pig. J Physiol. 1988;403:621-40. doi: 10.1113/jphysiol.1988.sp017268. PMID: 2855346.

42. Wang X, Wang X, Gu Y, Wang T, Huang C. Wenxin Keli attenuates ischemia-induced ventricular arrhythmias in rats: Involvement of Ltype calcium and transient outward potassium currents. Mol Med Rep. 2013;7(2):519-24. doi: 10.3892/mmr.2012.1195. PMID: 23174802.

43. Wang SY, Clague JR, Langer GA. Increase in calcium leak channel activity by metabolic inhibition or hydrogen peroxide in rat ventricular myocytes and its inhibition by polycation. J Mol Cell Cardiol. 1995;27(1):211-22. doi: 10.1016/s0022-2828(08)80020-x. PMID: 7760345.

44. Balnave CD, Vaughan-Jones RD. Effect of intracellular pH on spontaneous Ca2+ sparks in rat ventricular myocytes. J Physiol. 2000;528 Pt 1:25-37. doi: 10.1111/j.1469-7793.2000.00025.x. PMID: 11018103.

45. Kaplan P, Hendrikx M, Mattheussen M, Mubagwa K, Flameng W. Effect of ischemia and reperfusion on sarcoplasmic reticulum calcium uptake. Circ Res. 1992;71(5):1123-30. doi: 10.1161/01.res.71.5.1123. PMID: 1394874.

46. Hill JL, Gettes LS. Effect of acute coronary artery occlusion on local myocardial extracellular K+ activity in swine. Circulation. 1980;61(4):768-78. doi: 10.1161/01.cir.61.4.768. PMID: 7357719.

47. Pu J, Boyden PA. Alterations of Na+ currents in myocytes from epicardial border zone of the infarcted heart. A possible ionic mechanism for reduced excitability and postrepolarization refractoriness. Circ Res. 1997;81(1):110-9. doi: 10.1161/01.res.81.1.110. PMID: 9201034.

48. Baba S, Dun W, Cabo C, Boyden PA. Remodeling in cells from different regions of the reentrant circuit during ventricular tachycardia. Circulation. 2005;112(16):2386-96. doi: 10.1161/CIRCULATIONAHA.105.534784. PMID: 16203911.

49. Aggarwal R, Boyden PA. Diminished Ca2+ and Ba2+ currents in myocytes surviving in the epicardial border zone of the 5-day infarcted canine heart. Circ Res. 1995;77(6):1180-91. doi: 10.1161/01.res.77.6.1180. PMID: 7586231.

50. Aggarwal R, Boyden PA. Altered pharmacologic responsiveness of reduced L-type calcium currents in myocytes surviving in the infarcted heart. J Cardiovasc Electrophysiol. 1996;7(1):20-35. doi: 10.1111/j.1540-8167.1996.tb00457.x. PMID: 8718981.

51. Lue WM, Boyden PA. Abnormal electrical properties of myocytes from chronically infarcted canine heart. Alterations in Vmax and the transient outward current. Circulation. 1992;85(3):1175-88. doi: 10.1161/01.cir.85.3.1175. PMID: 1371431.

52. Jiang M, Cabo C, Yao J, Boyden PA, Tseng G. Delayed rectifier K currents have reduced amplitudes and altered kinetics in myocytes from infarcted canine ventricle. Cardiovasc Res. 2000;48(1):34-43. doi: 10.1016/s0008-6363(00)00159-0. PMID: 11033106.

53. Di Diego JM, Antzelevitch C. Ischemic ventricular arrhythmias: experimental models and their clinical relevance. Heart Rhythm. 2011;8(12):1963-8. doi: 10.1016/j.hrthm.2011.06.036. PMID: 21740880.
